# ClinVar and HGMD genomic variant classification accuracy has improved over time, as measured by implied disease burden

**DOI:** 10.1186/s13073-023-01199-y

**Published:** 2023-07-13

**Authors:** Andrew G. Sharo, Yangyun Zou, Aashish N. Adhikari, Steven E. Brenner

**Affiliations:** 1 Biophysics Graduate Group, University of California, Berkeley, CA 94720 USA; 2 Center for Computational Biology, University of California, Berkeley, CA 94720 USA; 3 Department of Ecology and Evolutionary Biology, University of California, 124 Biomed Building, 1156 High St., Santa Cruz, CA 95064 USA; 4 Department of Plant and Microbial Biology, University of California, 461 Koshland Hall, Berkeley, CA 94720 USA; 5 Currently at: Department of Clinical Research, Yikon Genomics Company, Ltd., Shanghai, China; 6 Currently at: Illumina, Foster City, CA 94404 USA

**Keywords:** ClinVar, HGMD, Pathogenic variant, Variant curation, Variant classification, Inborn errors of metabolism

## Abstract

**Background:**

Curated databases of genetic variants assist clinicians and researchers in interpreting genetic variation. Yet, these databases contain some misclassified variants. It is unclear whether variant misclassification is abating as these databases rapidly grow and implement new guidelines.

**Methods:**

Using archives of ClinVar and HGMD, we investigated how variant misclassification has changed over 6 years, across different ancestry groups. We considered inborn errors of metabolism (IEMs) screened in newborns as a model system because these disorders are often highly penetrant with neonatal phenotypes. We used samples from the 1000 Genomes Project (1KGP) to identify individuals with genotypes that were classified by the databases as pathogenic. Due to the rarity of IEMs, nearly all such classified pathogenic genotypes indicate likely variant misclassification in ClinVar or HGMD.

**Results:**

While the false-positive rates of both ClinVar and HGMD have improved over time, HGMD variants currently imply two orders of magnitude more affected individuals in 1KGP than ClinVar variants. We observed that African ancestry individuals have a significantly increased chance of being incorrectly indicated to be affected by a screened IEM when HGMD variants are used. However, this bias affecting genomes of African ancestry was no longer significant once common variants were removed in accordance with recent variant classification guidelines. We discovered that ClinVar variants classified as Pathogenic or Likely Pathogenic are reclassified sixfold more often than DM or DM? variants in HGMD, which has likely resulted in ClinVar’s lower false-positive rate.

**Conclusions:**

Considering misclassified variants that have since been reclassified reveals our increasing understanding of rare genetic variation. We found that variant classification guidelines and allele frequency databases comprising genetically diverse samples are important factors in reclassification. We also discovered that ClinVar variants common in European and South Asian individuals were more likely to be reclassified to a lower confidence category, perhaps due to an increased chance of these variants being classified by multiple submitters. We discuss features for variant classification databases that would support their continued improvement.

**Supplementary Information:**

The online version contains supplementary material available at 10.1186/s13073-023-01199-y.

## Background

Rare genetic diseases may affect as many as 1 in 20 Americans [1], but a definitive diagnosis is sometimes elusive [2]. In the past decade, exome and genome sequencing have improved the diagnostic rate for undiagnosed rare genetic diseases by 3- to fourfold over previously established methods [2–4]. Identifying the causal variant(s) through sequencing can inform disease management by altering treatment, predicting disease progression, and informing risk to other family members including reproductive planning [5, 6]. However, identifying causal variants can be challenging. Clinicians must objectively weigh many sources of evidence to determine if a variant explains the proband phenotypes. Indeed, the majority of individuals with a suspected rare genetic disease remain undiagnosed after exome or genome sequencing [2, 7].

To standardize the classification of variants, in 2015, the American College of Medical Genetics and Genomics (ACMG) and the Association for Molecular Pathology (AMP) developed guidelines to unify norms across clinical laboratories [8]. Since then, a growing number of laboratories have adopted these guidelines [9]. As familiarity with the guidelines has grown, variant classification concordance across laboratories has increased from 71% in 2016 to 84% in 2020 [10, 11]. These variant classification guidelines draw from several specialized research areas including population genetics, human gene isoforms, protein structure and function, and computational predictions of variant impact [12]. While these specialties have all made essential contributions to variant classification, perhaps no resource has been more valuable than the creation of diverse databases of allele frequencies, which are used to identify variants that are too common to cause a rare disease. In 2012, the Exome Sequencing Project created the first large-scale database of exonic allele frequencies that included samples from both European Americans and African Americans [13]. In 2015, phase 3 of the 1000 Genomes Project (1KGP) became available, providing genome-wide alleles from thousands of global genomes [14]. This was quickly followed by progressively larger and more diverse databases, including ExAC [15], gnomAD [16], and ALFA (Phan et al.: ALFA: Allele Frequency Aggregator, unpublished). Here, we investigate trends in the accuracy of variant classification since 2014, during which these allele frequency resources grew tremendously.

Researchers communicate variant classifications through published articles and submissions to variant databases. Until recently, variants were primarily classified in locus-specific databases (LSDBs) that typically collected variants in a single gene. In an effort to standardize content and improve ease of access, many LSDBs used the same software, the Leiden Open Variation Database [17], and the Human Genome Variation Society collected LSDBs to form a database of LSDBs [18]. Authoritative reference resources such as OMIM [19], GeneReviews [20], and GeneTests [21] often included additional variant information. Today, there are two leading genome-wide variant databases of clinical interest: ClinVar [22] and the Human Gene Mutation Database (HGMD) [23]. HGMD began in 1996, is now a commercial product with subscriptions sold by Qiagen, and is curated directly from published literature by dedicated staff. A free version of HGMD is available that is several years out of date. HGMD labels disease-causing variants as either “disease-causing” (DM) or “likely disease-causing, but with additional uncertainty” (DM?). HGMD contains nearly 385,000 variants classified as DM or DM?. Following calls to create an open-access database [24], in 2013, the NIH created ClinVar, a free-to-access database (maintained by NCBI, currently with input from ClinGen) that accepts submissions from clinical laboratories, research groups, and specialized databases. Mendelian disease-causing variants are submitted to ClinVar as either Pathogenic (P), Likely Pathogenic (LP), or Risk Allele for non-Mendelian effects. As of 2020, ~ 8000 users access ClinVar each day, and it currently contains P or LP classifications of nearly 218,000 variants [22]. By definition, P indicates a 99% chance of pathogenicity [25], and LP indicates a 90% chance of pathogenicity [8].

ClinVar and HGMD employ different strategies to reach the same goal: accurate variant classification. The largest volume (> 93%) of submissions to ClinVar come from clinical laboratories that typically use standardized interpretation guidelines to classify variants for pathogenicity. In addition to cataloging pathogenic variants, ClinVar also catalogs variants classified as benign or uncertain. HGMD curates information directly from publications, which may include experimental assays of variant function [23], but does not record variants reported as benign or uncertain. These databases are rapidly growing. Since 2017, the number of unique ClinVar variants has doubled, and HGMD variants have grown by 50%.

Several studies have attempted to assess the accuracy of cataloged variants using large sequencing cohorts of healthy individuals [26–30]. Two of the earliest studies searched for variants classified as pathogenic in individuals sequenced in a population database created by the 1000 Genomes Project (1KGP) [14]. These researchers identified individuals in 1KGP who were homozygous for one or more recessive variants classified as pathogenic (henceforth, “indicated affected individuals”). Surprisingly, these two studies found that most individuals harbored multiple homozygous variants that were cataloged by HGMD to cause early-onset disease. However, individuals in 1KGP were all over 18 years of age and healthy enough to sign a consent form. Certainly, 1KGP individuals are not expected to be enriched for disease, yet these studies found that the implied rates of disease were higher in 1KGP than the known disease prevalence. There are two plausible explanations for this discrepancy. The first is that many benign variants were misclassified as pathogenic, which the authors concluded [28, 29]. An alternative explanation is that some Mendelian diseases have been underdiagnosed. Since this is true for some disorders [31], we chose to analyze a subset that is screened for at birth and is likely not substantially underdiagnosed (see the “Methods” section). With this modification, we believe that most, and likely all, of indicated affected individuals are not affected by a disease, and rather the classified pathogenic variants they harbor were misclassified (see caveats below). A similar approach has also been used to investigate ClinVar variants, which a 2018 study showed imply disease prevalence much higher than the recorded prevalence for several clinically actionable or rare disorders [26]. Using orthogonal methods, researchers have identified variant features that are associated with the correct classification. Specifically, they have found that recently curated variants, with lower minor allele frequency (MAF), with multiple concordant submissions, and submitted by clinical laboratories, are more likely to be correctly classified [30, 32].

Since many variants are found principally in a single ancestral population, misclassification can lead to disparities in variant interpretation and clinical care. Indeed, one study determined that variants erroneously associated with sudden heart failure were found at higher allele frequency in Black Americans than in White Americans [33]. Fortunately, these misclassified variants were eventually corrected. However, until erroneously classified variants are corrected, which may take years, probands who harbor these variants may undergo inappropriate medical care. Furthermore, misclassified variants can have effects beyond the clinical care of individuals with those variants, since cataloged pathogenic variants can influence novel variant classification. In the ACMG/AMP variant classification guidelines, two categories of evidence that support pathogenicity rely directly on cataloged variants: the same amino acid change as an established pathogenic variant (PS1) and a different amino acid change at the same residue as an established pathogenic variant (PM5). Misclassified variants can also have indirect effects through the ACMG/AMP guidelines’ consideration of variant impact predictors, which contribute supporting evidence (PP3, BP4). Since many variant impact predictors are trained or are validated on cataloged variants [34–37], their predictions may be influenced by misclassified variants. In the worst case, a researcher following the ACMG/AMP guidelines may be misled by misclassified variants to incorrectly classify a novel variant, either by using misclassified variants as direct evidence (PS1, PM5) or indirectly though variant impact predictors that were trained on misclassified variants (PP3, BP4). Such an event would propagate existing variant misclassifications and possibly reinforce disparities.

Variant databases have taken different approaches to address misclassifications. ClinVar introduced a star system to indicate the review status of a variant classification, in which a variant gains credibility when assertion criteria are provided, multiple submitters concur, or a classification comes from experts in the field who follow gene-specific classification guidelines [38]. These review stars are distinct from the actual classification or probability of pathogenicity. For example, if multiple submitters classify a variant as VUS with assertion criteria, it will have two stars, indicating consensus that the pathogenicity remains uncertain. Throughout our analysis, we assess variant reclassification, when a variant changes from one of the three major tiers (P/LP, VUS/Conflicting, or B/LB) to another major tier. To provide additional granularity, we also evaluate variant recategorization, which includes reclassification, as well as when a variant changes in the number of stars (such as P 1 star to P 2 stars). Wright et al. found that variants classified as pathogenic with more review stars were more likely to be truly pathogenic [30]. ClinGen has also supported the formation of variant curation expert panels—composed of clinical laboratory staff, clinicians, and researchers with expertise relevant to a disease gene—which can provide high-confidence variant classifications and resolve conflicting variant classifications. Currently, ClinVar contains just 41 genes in which 10 or more variants are reported as reviewed by an expert panel, out of more than 3000 genes associated with a monogenic disorder by OMIM [19]. Although expert panels are promising, they have so far contributed to a small fraction of ClinVar variant reclassifications. HGMD curators reclassify variants based on newly published evidence such as functional studies or population frequency, and their reclassification rate has been reported as similar to that of ClinVar [23, 39]. Here, we consider whether these reclassification efforts, in concert with improved resources, have reduced the number of apparently misclassified variants over time. We consider variants in a subset of well-studied genes with highly penetrant phenotypes.

Inborn errors of metabolism (IEMs) are a group of rare, primarily recessive, or X-linked monogenic disorders caused by defects in a metabolic enzyme or its cofactors. Newborns in most developed countries are screened for IEMs using blood metabolites. Untreated, many of these screened IEMs are highly penetrant and lead to metabolite accumulation that often causes irreversible disability or death. They are thus a model system for identifying false positives in variant databases, as they should not be present as pathogenic genotypes in healthy individuals. While many screened IEMs are debilitating or fatal in childhood unless treated, there are notable exceptions. For example, our screened IEMs include short-chain Acyl CoA dehydrogenase deficiency (SCADD; associated with *ACADS*) and hyperprolinemia type I (HPI; associated with *PRODH*), both of which often do not yield symptomatic disease [40, 41]. Additionally, our screened IEMs include ornithine transcarbamylase deficiency (OTCD; associated with *OTC*) and glutaric acidemia type II (GAII; primarily associated with *ETFDH*), both of which are often seen in late-onset forms which may not result in outward symptoms until adulthood [42, 43].

Because screened IEMs are systematically identified in the population, their maximum possible incidence is generally known, and there has been a greater opportunity to identify and catalog the genetic variants that cause these diseases. Indeed, one recent study found potential benefits to screening newborns for IEMs using exome sequencing alongside mass spectrometry, the current standard for screening [44]. However, these researchers found it necessary to manually curate dozens of variants cataloged in ClinVar or HGMD for which the MAF was higher than expected for a rare disorder. Out of 60 variants with MAF > 0.1%, they deemed 41 were not reportable due to insufficient published evidence for pathogenicity.

Variants with a MAF greater than expected from disease incidence are addressed in the 2015 ACMG/AMP variant classification guidelines [8] under the BA1 evidence for benign variants. These guidelines recommend that a MAF > 5% in 1KGP, ExAC (now superseded by gnomAD), or the Exome Sequencing Project (ESP) may be considered stand-alone evidence that the variant is benign. In 2018, the guidelines for this classification were updated by Ghosh et al. to recommend that a MAF > 5% in any continental population dataset of at least 2000 alleles (with some additional constraints) is stand-alone evidence the variant is benign [45]. We have explored how implementing the original or revised guidelines impacts our results.

Here, we investigated how the degree of variant misclassification has changed over time in ClinVar and HGMD, using screened IEMs as a model system. Building on previously developed methods [28, 29], we used samples in the 1000 Genomes Project (1KGP) to identify individuals who harbor genetic variants that have been listed in ClinVar or HGMD as pathogenic. We identified more individuals than expected compared to the incidence of screened IEMs, which allowed us to assess the specificity of each database. Since we do not measure false negatives, we cannot assess the sensitivity of each database even though the balance between specificity and sensitivity is an important tradeoff to consider. We examined how the number of likely false-positive individuals indicated by ClinVar and HGMD changed over time, and we considered whether certain ancestry groups were over-represented.

## Methods

### Identifying indicated affected individuals in 1KGP

We used GRCh38 genotypes from 1KGP phase 3 [14] VCF files (downloaded on 14 November 2019) to identify individuals who harbor genotypes classified as pathogenic (defined as homozygous, hemizygous, or compound heterozygous) but who likely do not suffer from a screened IEM. 1KGP consists of 2504 individuals (661 of African ancestry, 347 of Latino ancestry, 504 of East Asian ancestry, 503 of European ancestry, and 489 of South Asian Ancestry). Individuals were over 18 years of age and healthy enough to sign a consent form. Ancestry was determined by superpopulation membership, as listed by the International Genome Sample Resource [46]. We created a curated list of 80 genes (Additional file 1: Table S1), associated with 48 IEMs screened by the California Newborn Screening Program [47] (henceforth, screened IEMs). These screened IEMs include some disorders where a large fraction of affected individuals is asymptomatic. In our analysis below, we identified several ClinVar variants in *PRODH*, associated with HPI. This condition is characterized by elevated levels of proline, and it is sometimes considered benign and asymptomatic [41]. However, there are reports of individuals with HPI who have a severe neurological impairment [48]. Additionally, recent long-term follow-up of patients with HPI suggests it results in impaired social skills, and there is evidence that deletions containing *PRODH* (and possibly variants in *PRODH*) contribute to schizophrenia risk [49–51]. Given the possible clinical phenotypes associated with this gene, we retained it in our analysis.

The population incidence of screened IEMs is approximately 1 in 3200 [52]. Thus, if the 2504 individuals sequenced in 1KGP were a random sample with unknown health status at birth, we would expect less than 1 individual to have a screened IEM. Given that most of the indicated affected individuals lived in countries without newborn screening programs before 1990 (Additional file 2: Table S2), they are unlikely to have been screened and treated early enough to prevent irreversible damage.

ClinVar GRCh38 variants were obtained from VCF files (downloaded on 8 January 2021) from the NCBI ClinVar FTP site [38]. VCF files were gathered from both archives 1.0 and 2.0 (starting with clinvar_20140401.vcf.gz and ending with clinvar_20201226.vcf.gz). Bcftools norm [53] was used to left-align and normalize indels. Only variants within our list of 80 genes were considered further. These methods are visually summarized in Additional file 3: Fig. S1A. Variants that were listed as only somatic or variants with null alt alleles were not considered further. For ClinVar archive 1.0 variants, variants were assigned clinical significance using the following categories: “0,” VUS; “2,” Benign (B); “3,” Likely Benign (LB); “4,” Likely Pathogenic (LP); and “5,” Pathogenic (P). Variants were inferred to have Conflicting classifications when they had classifications in two or more of the following three categories: B or LB, VUS, P, or LP. For ClinVar archive 2.0 variants, variants were assigned clinical significance using the following categories: “Benign,” B; “Benign/Likely_benign,” B; “Likely_benign,” LB; “Uncertain_significance,” VUS; “Likely_pathogenic,” LP; “Pathogenic/Likely_pathogenic,” P; “Pathogenic,” P; and “Conflicting_interpretations_of_pathogenicity,” Conflicting. For variants with multiple classifications separated by commas, if exactly one of the classifications was in the above list of categories, the variant was assigned to that category (e.g., “Pathogenic,_risk_factor” would be assigned to P). Due to inconsistencies in review star annotation in archive 1.0 files before June 15, 2015, review stars were not considered before this date. For archive 1.0 files after June 15, 2015, “no_assertion_criteria_provided,” “no_assertion_provided,” “not,” “no_criteria,” and “no_assertion” were grouped as 0 review stars; “criteria_provided,” “conf,” and “single” were grouped as 1 review star; and “_multiple_submitters,” “_no_conflicts”, and “mult” were grouped as 2 review stars. For all archive 1.0 files, review stars were assessed manually for variants with an inferred pathogenic genotype in 1KGP. For archive 2.0 variants, “no_assertion_criteria_provided,” “No_assertion_provided,” and “no_interpretation_for_the_single_variant” were grouped as 0 review stars; “criteria_provided,” “_single_submitter,” and “_conflicting_interpretations” as 1 review star; and “_multiple_submitters,” “_no_conflicts,” and “reviewed_by_expert_panel” as 2 + review stars. We note that historically, pathogenic OMIM variants were initially uploaded to ClinVar as “single submitter” and later systematically recategorized to “no assertion criteria provided.” Our recategorization results below suggest this event has been masked by subsequent submissions that also led to recategorization. In calculating indicated affected individuals for each year, we reported the maximum number of individuals with an inferred pathogenic genotype at any time in that year. In our analysis of 1KGP-indicated affected individuals, ClinVar submissions were removed from consideration if the submitted condition was not a screened IEM (e.g., schizophrenia). Submissions for which the condition was “not provided” were included in our analysis. For all other analyses, it was not feasible to check the submitted condition of variants.

HGMD variants were obtained from privately archived versions of HGMD 2014.1 and 2016.2, and a recently accessed version of 2020.3 through Qiagen Digital Insights HGMD Professional. Only SNVs classified at least once as “DM” or “DM?” within our list of 80 screened IEM genes were considered further. There were a handful of variants with two classifications, and these were assigned the more severe classification.

In our analysis using the 2015 BA1 guidelines, variants with a global MAF > 5% in 1KGP, the Exome Sequencing Project (ESP6500SI-V2), or gnomAD v2.1 exomes were removed from consideration. In our analysis using the 2018 BA1 guidelines, variants with a global MAF > 5% in 1KGP or ESP or a MAF > 5% in any gnomAD exome continental population were removed.

Ensembl Variant Effect Predictor with custom annotations was used to annotate the 1KGP VCF with all features. For rapid I/O of VCFs, we used cyvcf2 [54]. To identify when the ancestry composition of indicated affected individuals (aggregated across all screened IEMs) was significantly different from the ancestry composition of 1KGP or gnomAD, we first performed a two-sided Fisher’s exact test on a 5 × 2 contingency table that included the five continental populations (African, Latino, East Asian, European, South Asian), using fisher.test in the R “stats” package [55]. When the expected count for every population was greater than 40, we instead performed a Pearson’s chi-squared test using chisq.test to reduce computation time. For those global analyses that showed significant deviation from the 1KGP database ancestry composition, we performed individual tests to identify the significantly skewed population. These individual tests were performed using a one-sided Fisher’s exact test on a 2 × 2 contingency table as described above. To correct for multiple tests, we used a 5% significance threshold with Bonferroni correction for 222 tests, yielding a *p*-value threshold of 2.2 × 10^−4^. We determined 222 tests by calculating the total number of tests performed across all figures (including supplementary figures), which were typically 1 Fisher’s exact test per bar, with an additional 5 tests per bar when the Fisher’s exact test was significant. Bars that had zero height were not tested. Odds ratios and 95% confidence intervals were determined using two-sided Fisher’s exact tests as described above.

### Variant recategorization in ClinVar and HGMD

We next compared the degree of variant recategorization in ClinVar and HGMD, and we also quantified how ClinVar variant recategorization differs by ancestry. ClinVar and HGMD variants were filtered as described above. VEP [56] was used to annotate each variant with its gnomAD v2.1 exomes MAF. In order to identify recategorizations of review stars, for each time point available for ClinVar, each variant was classified into one of the following categories: B/LB 3 stars, B/LB 2 stars, B/LB 1 star, B/LB 0 stars, VUS, Conflicting, P/LP 0 stars, P/LP 1 star, P/LP 2 stars, or P/LP 3 stars. At each time point available for HGMD, each variant was classified into one of the following categories: DM, DM?, DFP, DP, or R. These categories are defined in Stenson et al. [23]. HGMD variants that were removed from the database were classified as R. Variants categorized in any other category (such as “not provided”) and all ClinVar variants prior to June 15, 2015, were not considered. To create Fig. 2A, B, for each variant, we considered only its first category chronologically (typically its category when first entered into the database) and its last category chronologically. These methods are visually summarized in Additional file 3: Fig. S1B.

Next, for each ClinVar variant, we used gnomAD v2.1 exomes to determine the ancestry group in which it occurs at the highest MAF. For the five continental ancestries we considered, gnomAD v2.1 exomes consist of 106,814 individuals (8128 of African ancestry, 17,296 of Latino ancestry, 9197 of East Asian ancestry, 56,885 of non-Finnish European ancestry, and 15,308 of South Asian ancestry). These samples were aggregated primarily from case–control studies of common adult-onset diseases [16]. To reduce bias from the unequal number of individuals in each ancestry group in gnomAD, all ancestry-specific MAFs below 6.152 × 10^−5^ (the smallest possible MAF in African ancestry, which has the smallest number of individuals in gnomAD) were set to zero. Next, each variant was assigned to the ancestry with the highest MAF. Variants with zero MAF in all ancestries were not considered further.

For each variant, we recorded all recategorizations it underwent. To avoid variants submitted prior to the introduction of review stars, only ClinVar reclassifications after June 15, 2015, were considered. ClinVar GRCh38 VCF files (as described above) were used to identify recategorizations. Recategorizations were considered every month. Since more recent ClinVar VCFs were archived weekly, these were downsampled to approximate monthly archives. The removal of a ClinVar variant from the database was not considered a recategorization. If a variant re-entered into ClinVar under a new category, it was considered recategorized.

Variant reclassifications were grouped into two categories: increasing confidence and decreasing confidence. Increasing confidence was defined as Conflicting or VUS to P/LP or B/LB with any number of stars. Decreasing confidence was defined as P/LP or B/LB with any number of stars to Conflicting or VUS. Variants were grouped by these categories, colored by assigned ancestry (see above), and visualized using Floweaver [57], resulting in Fig. 2C, E.

To correct for bias caused by the possible overrepresentation of some ancestries in ClinVar, for each ancestry, we calculated the number of variants in each category. The number of variants per category was calculated for every month, yielding a measure we call variant-months. A variant-month is a measure of both the number of variants and how long they have been in ClinVar. For example, 2 variants classified in ClinVar for a month are 2 variant-months, and 1 variant classified in ClinVar for 2 months is also 2 variant-months (see Additional file 4: Supplementary text 3B for a detailed example). For each ancestry, we analyzed its assigned variants to determine how many variant-months were cataloged for each category between June 15, 2015, and December 31, 2020. The differences in variant-months between ancestries reflect differences in genetic diversity as well as ClinVar submission bias. These variant-months are used to normalize comparisons across ancestries which we report in reclassifications per variant-month. In normalizing a reclassification category (increasing confidence or decreasing confidence), we divide the number of reclassifications by the variant-months of the source category. For example, if we wanted to compare increasing confidence across ancestries, then for each ancestry, we would calculate the number of reclassifications with increasing confidence among variants assigned to that ancestry and divide that by the variant-months of the source category, in this case, VUS and Conflicting variants. 95% confidence intervals were calculated for each ancestry group as ± 1.96*sqrt(p*(1 − p)/*n*) where *p* is reclassified variants/variant-months of source variants and *n* is variant-months of source variants. To identify significant reclassification rate differences between ancestries, we first performed a two-sided Fisher’s exact test on a 5 × 2 contingency table (as described above) containing the number of reclassifications and the number of variant-months of the source category for each ancestry. We then performed one-sided Fisher’s exact tests on 2 × 2 tables (as described above) for each possible pair of ancestries.

## Results

### Decrease over time in 1KGP individuals indicated as affected by Select ClinVar variants, indicating reduction in variants misclassified as P/LP with ≥ 1 review star

**Fig. 1:**
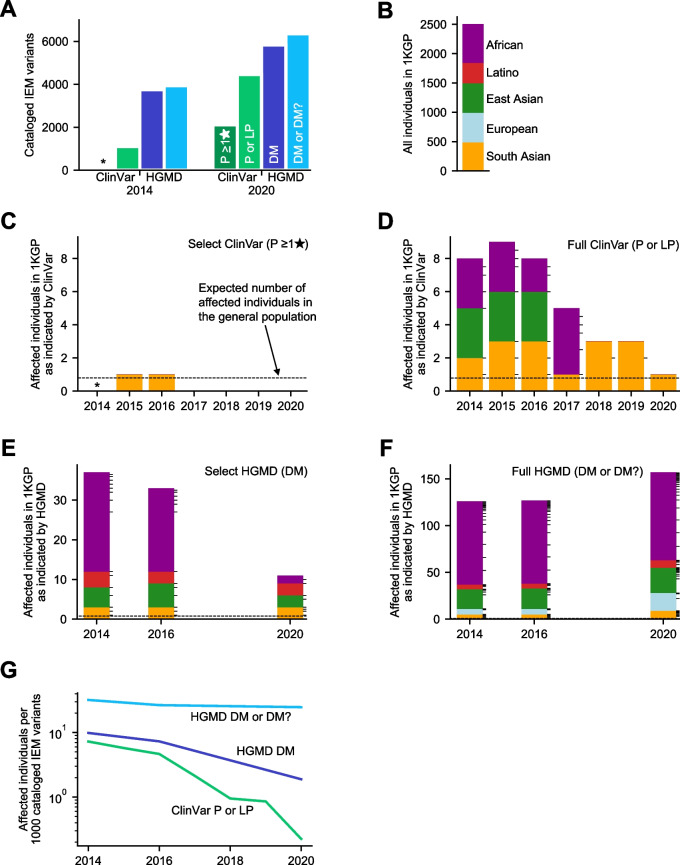
Number of 1KGP individuals indicated affected for screened IEMs by ClinVar or HGMD over time. **A** Number of screened IEM variants present in ClinVar or HGMD in 2014 and 2020. **B** Ancestry composition of individuals in 1KGP. **C**–**F** Bars are colored by ancestry as shown in **B**. Tick marks on bars cluster individuals by the variant classified as pathogenic that they harbor. Dashed black lines indicate the aggregate population incidence of screened IEMs. **C** The number of 1KGP individuals with an implied pathogenic genotype for a variant in Select ClinVar, defined as variants with a P classification with at least 1 review star. Variants that also have conflicting classifications (with VUS or B/LB) with 1 or more review stars are removed. **D** The number of 1KGP individuals with an implied pathogenic genotype for a variant in Full ClinVar, defined as variants with a P or LP classification. Variants that also have conflicting classifications (with VUS or B/LB) are removed. **E** The number of 1KGP individuals with an implied pathogenic genotype for a variant in Select HGMD variants, defined as variants classified as DM. 2014, 2016, and 2020 are shown because they are the years for which we have archived HGMD data. **F** The number of 1KGP individuals with an implied pathogenic genotype for a variant in Full HGMD variants, defined as variants classified as DM or DM?. **G** The number of affected individuals relative to the number of variants classified in each variant set. This approximates a false-positive rate, which has fallen over time for each database. *Data not available because the existing review star framework was not in place until 2015

We analyzed ClinVar-screened IEM variants submitted between April 2014 and December 2020 and first examined a Select subset based on review stars (see the “Methods” section). This Select subset included P variants with 1 or more review stars (indicating the submitter included assertion criteria), which consisted of 2118 variants in 2020 (Fig. 1A). In accordance with the 2015 ACMG/AMP BA1 guidelines, we removed variants with a MAF that reached the threshold for classification as stand-alone benign (global MAF > 5% in 1KGP, gnomAD, or ESP). This resulted in the removal of a single variant with 1 review star and a global MAF of ~ 5% (Additional file 5: Table S3). We later discuss applying the 2018 BA1 guidelines. To identify individuals who harbored inferred pathogenic genotypes of these Select ClinVar variants, we used the 1KGP database. 1KGP includes 2504 individuals that are drawn approximately evenly from 5 continental populations (Fig. 1B). We considered individuals to be indicated affected if they were homozygous, hemizygous, or compound heterozygous for one or more Select variants. We found a single indicated affected individual, with South Asian ancestry, who was homozygous for a P variant in *ACADS* (NM_000017.4:c.1108A > G) added to ClinVar in 2015, which was reclassified as Conflicting by 2017 (Fig. 1C; Table 1). There have since been zero indicated affected individuals through 2020.

**Table 1:** Subset of ClinVar variants seen in a pathogenic genotype in 1KGP. See Additional file 6: Table S4 for the full list of ClinVar variants. NC indicates no assertion criteria were provided by the submitter

Chr	Position in GRCh38	Ref	Alt	Gene, transcript ID	cDNA, protein	No. of hom or hemi in 1KGP	No. of comp het in 1KGP	1KGP sample ID(s)	First pathogenic submission: submitter, date, interpretation, evidence	Consensus interpretation as of December 2020	Submitted interpretations
9	130,458,549	G	T	ASS1, NM_054012.4	c.323G > T R108L	1	1	NA19030 NA19395	OMIM, April 2014, Pathogenic NC, heterozygous variant in affected individual	Conflicting Interpretations of Pathogenicity	1 Pathogenic NC 1 VUS 4 Likely Benign 1 Benign 1 Benign NC
12	109,561,798	C	T	MMAB, NM_052845.4	c.403G > A p.A135T	1	0	HG03169	GeneReviews, February 2016, Pathogenic NC, seen in affected individuals	Conflicting Interpretations of Pathogenicity	1 Pathogenic NC 1 VUS 1 Benign 1 Benign NC
12	120,739,317	A	G	ACADS, NM_000017.4	c.1108A > G p.M370V	1	0	NA20878	GeneDx, August 2015, Pathogenic, clinical testing	Conflicting Interpretations of Pathogenicity	2 VUS 1 Likely Benign
X	38,367,361	G	A	OTC, NM_000531.6	c.148G > A p.G50R	1	0	NA21124	GenMed Metabolism Lab, April 2014, Pathogenic NC, identified in late-onset individual	Pathogenic, 0 stars	2 Pathogenic NC

### Decrease over time in 1KGP individuals indicated as affected by Full ClinVar variants, indicating reduction in variants misclassified as P/LP with any number of review stars

In addition to considering variants in our Select ClinVar set, clinical laboratories will also typically review P and LP variants with 0 review stars (no assertion criteria) to determine if they can identify sufficient evidence for pathogenicity to influence their own classifications. To analyze these variants, we next considered the Full dataset of ClinVar-screened IEM variants, which included P and LP variants with any number of review stars. We removed from consideration variants that fulfilled the 2015 BA1 criteria. This eliminated six variants from 2014 to 2020, with a median MAF in 1KGP of 12% (Additional file 5: Table S3). We searched for individuals in 1KGP who were indicated affected (Fig. 1D). In 2014, there were 8 indicated affected individuals, which increased to 9 in 2015, and declined to just 1 by 2020 (reclassification causes discussed below). Eleven variants played a role in the genotypes of these indicated affected individuals (Additional file 6: Table S4). We also considered whether P or LP variants led to a larger number of indicated affected individuals. However, due to the relatively small fraction of variants where all submitters classified it as LP, the results of considering only P variants were nearly identical to considering both P and LP (Additional file 3: Fig. S2). We did not observe any statistically significant skew in the ancestries of the 1KGP individuals who were indicated affected.

### Decrease over time in 1KGP individuals indicated as affected by Select HGMD variants, indicating reduction in variants misclassified as DM

Similar to our ClinVar analysis, we first examined a Select subset of HGMD variants. This subset included HGMD DM (disease-causing) variants in any screened IEM gene, which consisted of 5833 variants in 2020. We removed one variant that met the 2015 BA1 criteria in 2014 and 2016 with a MAF of 20%. We removed an additional variant in 2016 with a MAF of 50% (Additional file 5: Table S3). We investigated individuals in 1KGP who harbored Select HGMD variants, and we found 37 indicated affected individuals in 2014, caused by 16 variants (Fig. 1E). Repeating this analysis with Select HGMD classifications from December 2020, we found 11 indicated affected individuals in 1KGP (70% reduction from 2014) due to 9 variants (reclassification causes discussed below). Three of these 9 variants were added to HGMD after 2014.

### Increase over time in 1KGP individuals indicated as affected by Full HGMD variants, indicating rise in variants misclassified as DM/DM?

To gain a larger picture of potential variant misclassification in HGMD, we next considered the Full dataset of HGMD variants classified to likely cause disease, which included DM and DM? variants (henceforth, Full HGMD variants). We removed 5 variants that met the 2015 BA1 criteria in 2014, 4 variants in 2016, and 7 variants in 2020 (Additional file 5: Table S3). We investigated individuals in 1KGP who harbored Full HGMD variants. In 2014, there were 126 indicated affected individuals in 1KGP due to 20 DM and 12 DM? variants (Fig. 1F). This increase in the number of DM variants compared to our Select analysis is due entirely to compound heterozygotes consisting of one DM variant and one DM? variant. Unexpectedly, we found indicated affected individuals increased over time, with 157 individuals in 2020, due to 17 DM and 27 DM? variants. These include 7 DM? and 4 DM variants that were added to HGMD since 2014.

### Ancestry skew discovered in 1KGP individuals indicated as affected by HGMD variants

These indicated affected individuals are not only a barometer for changes in potentially misclassified variants, but they can also inform whether particular ancestry groups are more likely to be affected by variant misclassifications. Considering Select HGMD variants in 2014, African ancestry individuals were significantly more likely to be indicated affected (Fig. 1E). While 26.4% of individuals in 1KGP are of African ancestry (Fig. 1B), 25 out of 37 (67.6%) of indicated affected individuals had African ancestry, which is significantly more than expected by chance (*p* < 10^−6^) and indicates an odds ratio of 5.8 for African ancestry individuals (95% CI: 2.8–12.8). By 2020, no populations were significantly skewed. Notably, in 2014, 2016, and 2020, no European ancestry individuals were indicated affected. When considering Full HGMD variants, we found that 89 out of 126 indicated affected individuals in 2014 and 94 out of 157 indicated affected individuals in 2020 were of African ancestry (both *p* < 10^−15^) (Fig. 1F). This translates to an odds ratio of 6.7 (95% CI: 4.5–10.2) in 2014 and 4.2 (95% CI: 3.0–5.9) in 2020. Unlike the ancestry skew observed in Select HGMD variants, the ancestry skew in Full HGMD variants has persisted over time.

### In both ClinVar and HGMD, there has been a decrease over time in the number of indicated affected individuals per cataloged variant

Since each ClinVar or HGMD dataset contains a different number of cataloged IEM-associated variants (Fig. 1A), we developed a metric to enable a comparison of false-positive rate across datasets. For each available year, we calculated the number of indicated affected individuals in 1KGP divided by the number of cataloged variants. Although we cannot be certain that no individual in 1KGP has a screened IEM, this metric is a proxy for the false-positive rate of each database. In 2014, the Full ClinVar dataset indicated 7.3 affected individuals per 1000 cataloged P or LP variants (Fig. 1G). By 2020, this false-positive rate had decreased by 97%. We could not determine a meaningful false-positive rate for the Select ClinVar dataset due to the several years with zero indicated affected individuals. For Select HGMD variants, the false-positive rate decreased by 81%, with most of this decrease occurring between 2016 and 2020 (Fig. 1G). For Full HGMD variants, the false-positive rate decreased by 26% from 2014 to 2020 (Fig. 1G). It may seem surprising that the false-positive rate of Full HGMD variants is decreasing given the increase in indicated affected individuals over time (Fig. 1F). However, this decrease is due to the ~ 60% growth in cataloged variants between 2014 and 2020, which outweighed the growth in indicated affected individuals.

These three datasets have reduced the false-positive rate of their cataloged variants over time, yet false-positive rates currently differ greatly between them. As of 2020, Full ClinVar variants indicate 0.22 affected individuals per 1000 cataloged pathogenic variants, which is an order of magnitude lower than Select HGMD variants, which indicate 1.9 affected individuals per 1000 cataloged pathogenic variants (Fig. 1G). This, in turn, is an order of magnitude lower than Full HGMD variants, which indicate 25 affected individuals per 1000 cataloged pathogenic variants. We additionally compared the incidence of screened IEMs inferred from each database with the known incidence of screened IEMs (Additional file 4: Supplementary text 1B, 2A; Additional file 3: Fig. S3, S4). Our findings were largely consistent with our indicated affected individual results. Furthermore, we replaced 1KGP with gnomAD v3.0 genomes to assess the reproducibility of our results. Overall, our gnomAD analysis replicated all major findings from our 1KGP analysis (Additional file 4: Supplementary text 2C, F; Additional file 3: Figs. S5-S9).

### Most ClinVar variants contributing to an inferred pathogenic genotype have been reclassified

Between 2014 and 2020, 11 screened IEM variants in the Full ClinVar dataset were part of an inferred pathogenic genotype in at least one 1KGP individual (Additional file 6: Table S4). As of December 2020, 10 of these 11 variants have been reclassified in ClinVar to a non-pathogenic category. Eight variants were reclassified to Conflicting, 1 variant to VUS, and 1 variant to B/LB, while one variant remained classified as P with 0 review stars. These variants were present in 7 genes: *OTC* (3), *ASS1* (2), *PRODH* (2), *ACADS* (1), *MMAB* (1), *MMUT* (1), and *SLC22A5* (1). Variants within the same gene tended to be initially contributed by the same submitter. For example, GenMed Metabolism Lab submitted the first classification for all three variants in *OTC*, and OMIM first provided both *PRODH* variants. For each variant, we also recorded the submitter that contributed the first non-pathogenic classification but did not identify any patterns (Additional file 6: Table S4).

Among these 11 variants, we noticed a trend. Variants were initially submitted as P or LP when seen in an affected individual, even though there was limited evidence for pathogenicity. Most of these variants were submitted before review status stars were introduced to ClinVar. As more information became available, such as MAF, later submitters, most using defined criteria, classified these variants as VUS, B, or LB. One illustrative case is the variant NM_052845.4:p. Ala135Thr in *MMAB* (Table 1). Through a semi-automated process, this variant was extracted from a GeneReviews table to a ClinVar record in February 2016 as P with two articles referenced to support the classification [58, 59]. These articles from 2002 and 2006 describe two individuals with methylmalonic acidemia (MMA) cblB type who each harbor the same three unphased heterozygous variants: p.Ala135Thr, NM_052845.4:p.Arg191Trp, and NM_052845.4:p.Tyr219Cys. Both articles state the p.Ala135Thr variant was absent from their control samples, for which ancestry information was not provided. We now know the MAF of this variant in African ancestry individuals is approximately 1% in 1KGP and gnomAD exomes, corresponding to a disease incidence of 1 in 10,000 assuming complete penetrance. However, MMA cblB type occurs in less than 1 in 50,000 births and has not been seen at elevated levels in individuals of African ancestry [52]. This variant was observed in a homozygous state in an African ancestry male in 1KGP, who most likely did not have MMA cblB type, which is a neonatal-onset disorder that results in severe disability and sometimes death without treatment. To ensure that this genotype was not caused by errors in variant calling, we independently base-called all Select ClinVar variants, Full ClinVar variants, and Select HGMD variants present in a pathogenic genotype (Additional file 4: Supplementary text 1A, 2B, Additional file 7: Table S5). Since the P submission, GeneDx used variant classification criteria to classify this variant as VUS, citing the relatively high variant frequency as evidence for benignity. Invitae (with criteria) and Natera (without criteria) have classified the variant as B. A plausible explanation for this history of classifications is inadequate information about this allele’s pertinent population frequency meant it was mistakenly associated with MMA in the two affected individuals.

As of 2020, there was a single 1KGP indicated affected individual. We examined the responsible ClinVar variant and found this variant could not be ruled out as disease-causing. GenMed Metabolism Lab submitted this variant, NM_000531.6:p.Gly50Arg in *OTC*, an X-linked gene, in 2014 and cited an article in which researchers found this variant in a male with late-onset Ornithine transcarbamylase deficiency (OTCD) but did not provide the age of onset [60]. OTCD has a variable age of onset, with the oldest reported proband 44 years old when disease onset began [42]. No other evidence supports pathogenicity. However, because the 1KGP hemizygous South Asian ancestry male (NA21124) has not yet reached the maximal age of onset, a pathogenic classification cannot be ruled out.

### Half of HGMD DM variants contributing to an inferred pathogenic genotype have been reclassified to DM?

In 2014, 16 Select HGMD variants contributed to an inferred pathogenic genotype in at least one 1KGP individual (Additional file 8: Table S6). By December 2020, 8 of these variants were reclassified to DM?, and an additional 3 DM variants were cataloged that contributed to an inferred pathogenic genotype. In total, we observed 19 Select variants in an inferred pathogenic genotype, which were present in 11 genes: *OTC* (4), *PAH* (3), *ASS1* (2), *CBS* (2), *CPT2* (2), *ACAD8* (1), *ACADS* (1), *ACADVL* (1), *SLC22A5* (1), *SLC25A13* (1), and *TAZ* (1). We did not evaluate the Full HGMD variants in detail, but we do note that of the 32 DM and DM? variants that contributed to an inferred pathogenic genotype in 2014, none was reclassified to a non-disease-causing category by 2020. Relationships between DM/DM? and P/LP variants are noted in Additional file 4: Supplementary text 2G.

HGMD rarely provides explanations for variant reclassification, so it is difficult to directly investigate why certain variants were reclassified. Instead, we examined the evidence for pathogenicity of the 19 Select variants identified in an inferred pathogenic genotype in 2014, 2016, or 2020. For each variant, we reviewed the articles cited by HGMD (Additional file 8: Table S6). According to the cited articles, researchers observed these variants in probands who were diagnosed with an IEM. None of the articles provided evidence for pathogenicity equivalent to the ACMG/AMP guidelines, which is not surprising given that most of the articles were published prior to 2015. Additionally, 14 out of 19 studies (74%) did not show any direct evidence for the functional effect of the variant, such as experimental assays of gene expression or enzymatic activity, and therefore did not conclusively assign pathogenicity to the variant (Additional file 8: Table S6). Assay absence was highly correlated with later reclassification from DM to DM?. Of the 4 variants classified as DM in 2014 for which assays were performed, all remained DM through 2020. Of the 12 variants for which no assay was performed, 8 were reclassified to DM? by 2020. Despite the predictive power of assay presence, the results of the assays were not always conclusive. For example, we found one 1KGP individual was homozygous for the variant NM_000017.4:c.1108A > G (Met370Val) in *ACADS*, which was cataloged by HGMD as DM (Table 2). Yet, the original article cited by HGMD indicates that the variant c.1108A > G has a much more mild effect on tetramerization than all other putatively pathogenic variants tested [61]. Similarly, functional assays of the variant NM_000071.3:c.1105C > T(Arg369Cys) in *CBS* in a yeast model indicated no effect on enzyme function in the article cited by HGMD [62] (Table 2).

**Table 2:** Subset of HGMD variants seen in a pathogenic genotype in 1KGP. See Additional file 8: Table S6 for the full list of HGMD variants

Chr	Position in GRCh38	Ref	Alt	Gene, transcript ID	cDNA, protein	No. of hom or hemi in 1KGP	No. of comp het in 1KGP	1KGP sample ID(s)	Variant-specific assay	Outcome	PubMed ID	HGMD 2014	HGMD 2020
12	120,739,317	A	G	ACADS, NM_000017.4	c.1108A > G p.M370V	1	0	NA20878	In vitro activity in mouse mitochondria	Very mild effect on protein misfolding	18523805	DM	DM
21	43,060,481	G	A	CBS, NM_000071.3	c.1105C > T p.R369C	0	2	HG02645 NA20289	Yeast model	No effect on enzyme function	9361025	DM	DM
X	38,381,417	C	T	OTC, NM_000531.6	c.374C > T p.T125M	1	0	NA19117	No	NA	8807340	DM	DM

### Applying expanded allele frequency stand-alone benign guidelines resolves ancestry skew in individuals indicated as affected by HGMD variants

We next considered how the use of the 2018 updated BA1 guidelines [45] changed the number of 1KGP individuals who were indicated affected. In accordance with the guidelines, we removed variants that had a MAF > 5% in any gnomAD exomes continental population. This had no effect on our analysis of Select or Full ClinVar variants (Additional file 3: Fig. S10A, B). Applying these guidelines to Select HGMD variants led to the removal of 1 variant in 2014 and 2016 (Additional file 9: Table S7; Additional file 3: Fig. S10C). We found that this reduced the Select HGMD indicated affected individuals by 15 African ancestry individuals in 2014 and 2016, while the 2020 individuals remained at 11. We next applied these guidelines to the Full HGMD variants, which led to the removal of 8 variants in all 3 years and reduced the number of indicated affected individuals by 75% in 2014 and 62% in 2020 (Additional file 3: Fig. S10D). Additionally, there was no remaining significant ancestry skew after correcting for multiple tests (see the “Methods” section). Overall, when we remove P/LP and DM/DM? variants according to the 2018 BA1 guidelines, the ClinVar and HGMD datasets had similar rates of false-positive individuals in 2014 and only recently have their rates diverged (Additional file 3: Fig. S10E).

### ClinVar variants are reclassified at a rate sixfold greater than those in HGMD

Our analysis of reclassified variants has so far considered only those variants which contributed to an inferred pathogenic genotype in 1KGP individuals. To identify broad trends in variant recategorization in ClinVar and HGMD, we considered all screened IEM variants that were recategorized in tier or review stars in ClinVar or HGMD between 2014 and 2020.

**Fig. 2:**
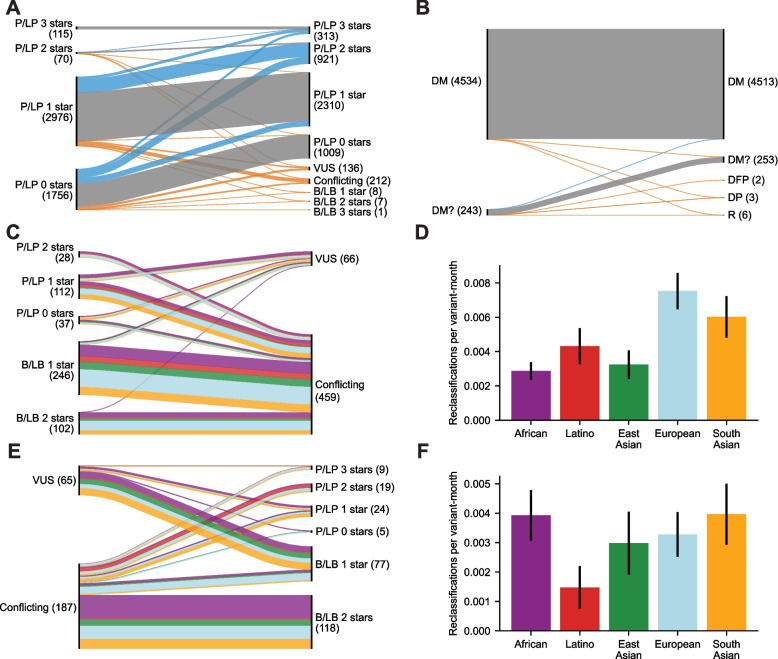
Variant reclassification in ClinVar and HGMD. **A** Reclassification paths of P/LP screened IEM ClinVar variants from 2014 (or first submission thereafter) to 2020, visualized in a Sankey plot in which line width represents the number of reclassified variants. Blue lines indicate increasing pathogenicity or review stars, orange lines indicate increasing benignity or reduced confidence of pathogenicity, and gray lines indicate no change. Numbers in parentheses provide variant counts of initial and final classifications for each category. **B** Reclassification paths of DM and DM? HGMD variants from 2014 to 2020. Disease-associated polymorphism (DP) and disease-associated polymorphism with additional functional evidence (DFP) are used to classify variants associated with disease but not necessarily disease-causing. Variants are retired (R) when they are found to no longer be associated with disease. **C** Reclassification paths of ClinVar variants from P/LP or B/LB to VUS or Conflicting. We plot only variants that could be assigned to a principal ancestry. Variant paths are colored by ancestry as in **D**. **D** Rate of reclassification of variants shown in **C** when normalized by the historical ancestry composition of variants in ClinVar. **E** Reclassification paths of ClinVar variants from VUS or Conflicting to P/LP or B/LB. **F** Rate of reclassification of variants shown in **E** when normalized by historical ancestry composition of variants in ClinVar

Out of 16,857 ClinVar variants, 3772 (22%) were recategorized between April 2014 and December 2020 (Additional file 3: Fig. S11). Of these recategorized variants, 28% were recategorized 2 or more times. To simplify our analysis, for each variant, we considered only the variant’s category when it first entered ClinVar and the variant’s category at the end of 2020. Of the 4917 P/LP variants in ClinVar between 2014 and 2020, we found 1655 (34%) were recategorized by the end of 2020 (Fig. 2A). Seventy-eight percent of these recategorizations were towards greater evidence for pathogenicity, and the remaining 22% were towards reduced evidence for pathogenicity (8% of all P/LP variants). The most common recategorization towards greater evidence for pathogenicity was from P/LP 1 star to P/LP 2 stars. The most common recategorization towards reduced evidence for pathogenicity was from P/LP 1 star to Conflicting.

HGMD-screened IEM variants were recategorized substantially less often than those in ClinVar. Out of 4777 variants classified as DM or DM? in 2014 or 2016, just 40 (0.8%) were recategorized. Seven of these recategorizations were from DM? to DM, and the remaining 33 were towards reduced evidence for pathogenicity (0.7% of all DM or DM? variants). The most common recategorization towards reduced evidence for pathogenicity was from DM to DM?. Between 2014 and 2020, only 6 DM or DM? variants were retired.

When considering variants recategorized towards reduced evidence for pathogenicity, we found that ClinVar variants were recategorized at a rate 11-fold greater than those in HGMD. We recognize this analysis is impacted by the greater number of available time samples and variant categories in ClinVar compared to HGMD, because every recategorization in HGMD is also a reclassification. However, when we repeat this analysis considering only ClinVar variants at time points for which HGMD data is available, while also collapsing ClinVar pathogenic variants to just 2 categories (P and LP), the difference is reduced but remains 11-fold to two significant figures. Because HGMD does not have a mechanism to classify variants as Conflicting, we repeated this analysis while also removing from consideration ClinVar variants reclassified to Conflicting. This revealed that ClinVar variants were reclassified sixfold more often than those in HGMD. Another relevant comparison is between ClinVar 0 star variants and HGMD, which also shows significantly greater reclassification in ClinVar (Fig. 2A).

### ClinVar variants common in European and South Asian individuals were more likely to be reclassified to a lower confidence category

In our earlier analysis, we identified ancestry skew in likely misclassified variants. Next, we investigate whether ancestry influences overall reclassification rates of variants in ClinVar. Historically, large-scale exome and genome sequencing projects (from which MAF is often derived) have undersampled non-European individuals [63, 64]. Thus, we suspected that non-European individuals may shoulder a larger burden of variants that were initially classified as P or LP due to uncertain MAF and later reclassified to be VUS or Conflicting. At the same time, we recognized that the largest ClinVar submitters are located in countries where a majority of the population has European ancestry. Consequently, variants common in European ancestry individuals may have a greater chance of being classified by multiple submitters which could lead to Conflicting classifications.

To distinguish which of these effects likely dominate in ClinVar, we determined whether variants present in specific ancestries were disproportionately likely to be reclassified. First, for each variant, we used gnomAD exomes to identify the continental ancestry group with the highest MAF, and we assigned the variant to that ancestry group. gnomAD exome MAFs were normalized to avoid bias from sample size differences between ancestries (see the “Methods” section). We first considered variants for which the classification was reduced in confidence, which includes P/LP and B/LB variants that were reclassified to VUS or Conflicting. For those variants that could be assigned to an ancestry, we visualized reclassifications using Sankey diagrams in which line width represents the number of reclassified variants, and lines were colored by ancestry (Fig. 2C). We observed that European ancestry variants were the largest group in most reclassification paths. However, this analysis did not account for the differences in ancestry composition of the variants submitted to ClinVar. To control for this potential bias, for each ancestry, we normalized by both the number of variants assigned to that ancestry and the duration in which they were in ClinVar which we measure in variant-months (see the “Methods” section). One variant-month is equivalent to a single variant classified in ClinVar for 1 month. We normalized only by variants that could have contributed to the reclassification (in this case, P/LP and B/LB). Controlling for the ancestry composition of variants in ClinVar, we found that variants for which European ancestry individuals had the highest MAF were reclassified towards greater uncertainty at a rate of ~ 0.8% per variant-month (Fig. 2D). This was approximately twice the rate of reclassification for variants for which African, East Asian, or Latino ancestries (all *p* < 8 × 10^−5^) had the highest MAF (Fig. 2D). This is consistent with our observation that among all variants classified in ClinVar, a larger fraction of European ancestry variants were classified as Conflicting (Additional file 3: Fig. S12). South Asian variants were also found to have elevated reclassification towards greater uncertainty of approximately 0.6% per variant-month, which is significantly higher than East Asian or African variants (both *p* < 2 × 10^−4^).

We also considered variants for which classification increased in confidence, which includes VUS or Conflicting variants that were reclassified to P/LP or B/LB. After visualizing these reclassifications with Sankey plots, we observed that in many reclassification paths, European variants were not the largest group (Fig. 2E), in contrast with reclassification paths towards less confidence. Indeed, when we normalized by the ancestry composition of variants in ClinVar, we found no significant difference between variants most common in African, East Asian, European, or South Asian ancestry, each of which was reclassified at ~ 0.3% per variant-month (Fig. 2F). The exception were variants most common in Latino ancestry, which were reclassified at ~ 0.1% per variant-month.

## Discussion

Variant databases are under continuous development and growth [22, 23]. Several studies have attempted to capture this progress at different snapshots in time, although these studies have generally looked at different database elements, making comparisons across time difficult [26–28, 65]. Here, we investigated not a single point in time but evaluated systematically the same disorders over 6 years across two different databases.

### Both ClinVar and HGMD have shown marked improvements in variant classification accuracy over time

In both databases, we observed a decrease over time in the number of 1KGP individuals indicated affected by an IEM. Based on the high temporal resolution the ClinVar archives afford, we can see this change was most pronounced in 2016 through 2018 after the establishment of the 2015 ACMG/AMP guidelines and coincident with allele frequency resources such as ExAC. We believe screened IEMs provide an informative lens that reveals broader database trends that may be representative of thousands of rare genetic disorders.

### The higher number of indicated affected individuals in HGMD relative to ClinVar likely reflects the different methods each database uses to source and catalog variants

Perhaps our most striking finding is the large difference between the number of affected individuals indicated by HGMD and ClinVar in 2020. However, this difference is not entirely surprising. The authors of published literature may not apply formal variant classification guidelines to their variants and in some cases imply pathogenicity by inclusion in tables of variants. HGMD states that its curation policy is “to err on the side of inclusion and enter a variant into the database even if its pathological relevance may be questionable” and uses DM? classifications for this purpose as well as frequency flags in its online interface [66]. Additionally, HGMD does not capture conflicting evidence from VUS or B/LB classifications and must contend with the heterogeneity of the literature. On the other hand, the clinical laboratories that contribute to ClinVar typically apply professional guidelines to classify variants and aim for consistent reporting for clinical use. An additional factor may be the increasing use of assertion criteria in variants contributed to ClinVar, which compels contributors to include consistent supporting evidence leading to a classification or provide a public contact for their records. In contrast, many journals (from which HGMD curates variants) do not require that variants be classified according to current clinical practice guidelines. Therefore, this analysis should not be seen as a duel between two competing databases, but rather a quantitative comparison between two distinct methods for sourcing and cataloging variants. These distinct methods led to the 100-fold difference between the false-positive rate of individuals indicated affected by Full ClinVar variants and Full HGMD variants, observed in both 1KGP (Fig. 1G) and gnomAD (Additional file 3: Fig. S5G). While the Full HGMD rate (~ 25 indicated affected individuals per 1000 cataloged variants) is still relatively low, our analysis allows us to quantify this difference in specificity between the two databases. It is possible that a clinical analysis using HGMD, which has more unique DM or DM? variants than ClinVar has unique P or LP variants, would result in a higher sensitivity analysis, but we are not able to assess false negatives in this study. Understanding the differences between these databases may be valuable to not only clinical researchers, but also to non-domain experts such as computational researchers, who sometimes use HGMD and ClinVar interchangeably to develop variant interpretation methods [67].

### Updated BA1 guidelines to classify common variants as benign remove lingering ancestry-specific false-positive variants

Due to founder mutations, individual IEM variants are often enriched in a single ancestry. However, when we consider the total burden of all screened IEMs, continental ancestry groups appear to be affected at similar rates [52]. We found that African ancestry individuals were disproportionately indicated affected in 2014 when HGMD Select variants were considered, but this skew was resolved by 2020. Yet, all of the DM variants causing the ancestry skew in 2014 were reclassified to DM?. Thus, when considering HGMD Full variants, we found that significant African ancestry skew remained. Encouragingly, when we applied the 2018 BA1 guidelines, we observed no significant ancestry skew among Full or Select HGMD variants. This suggests that much of the observed ancestry skew is due to population-specific common variants. This likely reflects the historical lack of African ancestry samples in large sequencing projects [68, 69]. HGMD in particular may be susceptible to these factors, since it catalogs variants directly from publications, including older literature that was written when common variants in African ancestry individuals were poorly characterized. When older studies are given the same credence as recent ones, these disparities are more likely to be perpetuated.

### Outlier “zombie” variants should be a priority for ClinVar to address

Among ClinVar variants that were reclassified, very rarely did the initial submitter change their classification, and instead nearly all were reclassified due to subsequent conflicting classifications that largely included assertion criteria. We carefully examined 10 ClinVar variants which previously contributed to an inferred pathogenic genotype but have since had new non-pathogenic classifications submitted. By 2020, eight of these variants were classified as Conflicting. For many variants, this is an accurate descriptor and reflects enduring disagreement among submitters. However, for some variants, this may be a byproduct of ClinVar’s current design—an archive of claims of variant significance without a mechanism to vet them. ClinVar calculates an overall classification of a variant based on aggregating all submissions. An older, incorrect classification is suppressed only if a submission with a higher review status is submitted. For example, if an incorrect submission is at a 0-star level (no assertion criteria), a newer submission with assertion criteria will override the classification. Likewise, an expert panel or professional guideline (3 or 4 stars) will override classifications on a variant with 0–2 stars review status. However, if submissions are of the same review status, the record remains Conflicting until the P or VUS submitter changes or retracts their submission. If a submitter is not responsive to requests to update entries from the broader community, then an older submission will persist in the aggregate classification calculated by ClinVar. Although this system has advantages (historical knowledge is not lost), it may also impede the resolution of variants and indicate conflict when there is a large consensus. For example, NM_054012.4:c.323G > T in *ASS1* is currently listed in ClinVar as Conflicting, yet it has the following classifications with at least 1 review star: 1 B, 4 LB, and 1 VUS. The VUS classification is from 2017, while the 5 B/LB classifications are more recent. Although researchers have found that older variant classifications tend to be less accurate, their influence persists [32]. This is even more true for HGMD, which predates ClinVar and thus also contains a large fraction of older classifications. Given the rapid increase in our ability to determine variant MAF and predict variant pathogenicity, even in the past 5 years, it may be reasonable to request ClinVar submitters refresh older outlier classifications or have ClinVar deprecate them to reduce the influence of incorrect “zombie” classifications that persist and lead to unnecessary conflicts. Regardless of the exact strategy, methods to confirm the validity of older outlier classifications will be valuable.

This work has several limitations. Among rare diseases, the variants associated with screened IEMs are unusually well-curated thanks to newborn screening programs. Thus, screened IEMs are not necessarily representative of many rare diseases. Furthermore, our primary analysis was limited by the comparably small size of 1KGP relative to the rarity of IEMs. At the same time, 1KGP has several advantages, including its approximately even representation of the 5 major continental ancestries and its open availability of genomes, which allowed us to identify individuals who are compound heterozygous for variants classified as pathogenic and to validate the quality of nearly all analyzed variants. These unique features give 1KGP enduring value. Both 1KGP and gnomAD lack representation from some populations, including the Middle East, Oceania, and much of the African continent. These gaps impede the identification of benign variants from their high allele frequency in these populations. As this problem afflicts both clinical laboratory variant classification and our analysis, it is possible that our results may not be applicable to such underrepresented populations. Our analysis was particularly sensitive to putatively misclassified variants on the X chromosome since we considered males who were hemizygous for a variant classified as pathogenic to be indicated affected. This explains the relatively high number of observed *OTC* and *TAZ* variants flagged by our analyses of ClinVar and HGMD, despite the extreme rarity of their associated disorders. Finally, since few ClinVar submitters provide detailed explanations for their classification, and HGMD does not provide detailed explanations for its classifications, for many variants, it is difficult to determine with confidence why classifications changed over time.

## Conclusions

We have investigated how the false-positive rate of ClinVar and HGMD variants has changed over time. Our results suggest that ClinVar has a lower false-positive rate than HGMD due to variant reclassification occurring in the past few years. We noted patterns in variant reclassification and found that variant classification guidelines and diverse allele frequency databases principally contributed to these reclassifications. In agreement with the lower false-positive rate of ClinVar variants, we found that variants classified as pathogenic in ClinVar are reclassified sixfold more often than those in HGMD, suggesting that misclassified variants are more readily reclassified in ClinVar than HGMD. We also discovered that variants common in European and South Asian individuals were significantly more likely to be reclassified from P/LP or B/LB to VUS or Conflicting. We conclude that although the allele frequency of variants common in European individuals has been known for longer, due to the increased chance they will be classified by multiple submitters, they are more often reclassified from a confident category to a less confident category in ClinVar. We anticipate that this work will be a valuable benchmark of the progress that has been made in variant interpretation, of interest to the individuals who maintain these databases, the clinical laboratories and researchers who use these databases regularly, and the computational researchers who use these databases for training and testing methods.

## Supplementary Information

**Additional file 1:** **Table S1.** list of screened IEM genes and their associated disorders.

**Additional file 2:** **Table S2.** list of indicated affected individuals ordered by 1KGP subpopulation, and whether newborn screening likely occurred for these individuals.

**Additional file 3:** **Supplementary Figs. S1-S12**.

**Additional file 4:** **Supplementary Texts 1A-3B**.

**Additional file 5:** **Table S3.** Variants removed due to 2015 BA1 guidelines.

**Additional file 6:** **Table S4**. ClinVar variants found in a pathogenic genotype in one or more predicted affected individuals from 1KGP.

**Additional file 7:** **Table S5.** Quality confirmation of Select ClinVar variants, Full ClinVar variants, and Select HGMD variants present in a pathogenic genotype.

**Additional file 8:** **Table S6.** HGMD variants found in a pathogenic genotype in one or more predicted affected individuals from 1KGP.

**Additional file 9:** **Table S7.** Variants removed due to 2018 updated BA1 guidelines.

## Data Availability

All datasets and code generated and analyzed during this study are available in the Dryad repository: (https://doi.org/10.6078/D1872X) [70], with the following exception. Data obtained from HGMD are available only by subscription, available at https://digitalinsights.qiagen.com/products-overview/clinical-insights-portfolio/human-gene-mutation-database/.
