# Supplementary material for: ClinVar and HGMD genomic variant classification accuracy has improved over time, as measured by implied disease burden
Source: Genome Med. 2023 Jul 13;15:51. doi: 10.1186/s13073-023-01199-y (PMC10347827; doi:10.1186/s13073-023-01199-y)
Supplement: Supplementary file 3 — Additional file 3: Supplementary Figs. S1-S12. [file 13073_2023_1199_MOESM3_ESM.docx]

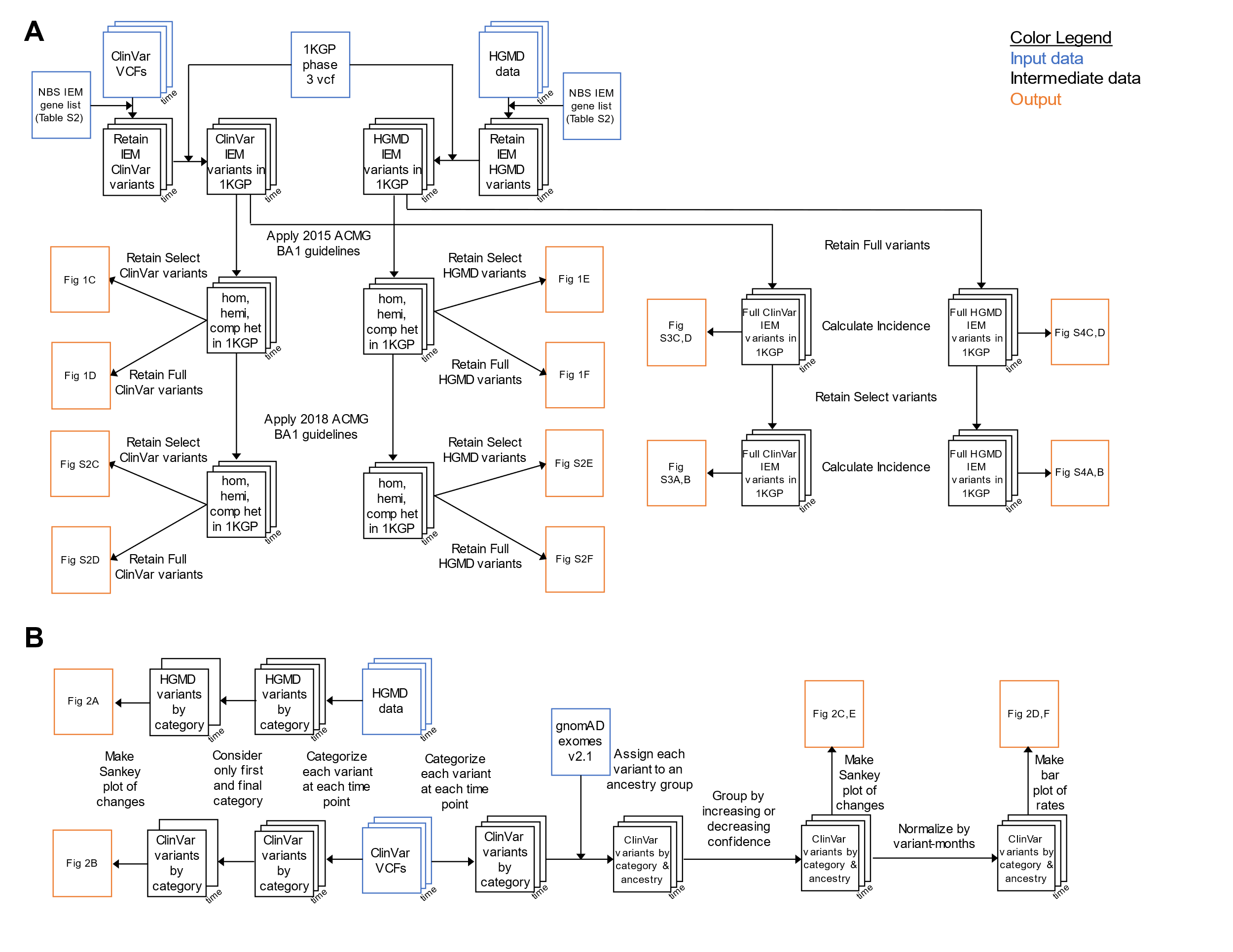


**Fig. S1.** (A) Data flow chart for figures depicting indicated affected individuals and incidence. (B) Data flow chart for figures depicting variant recategorization.

**A**

**
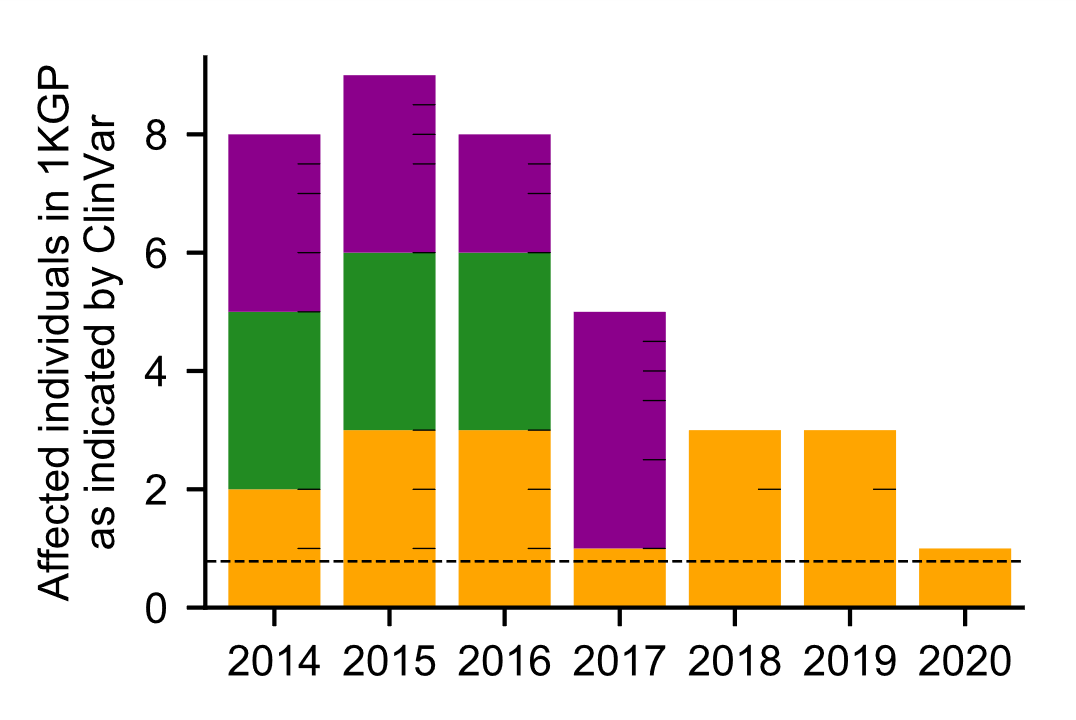

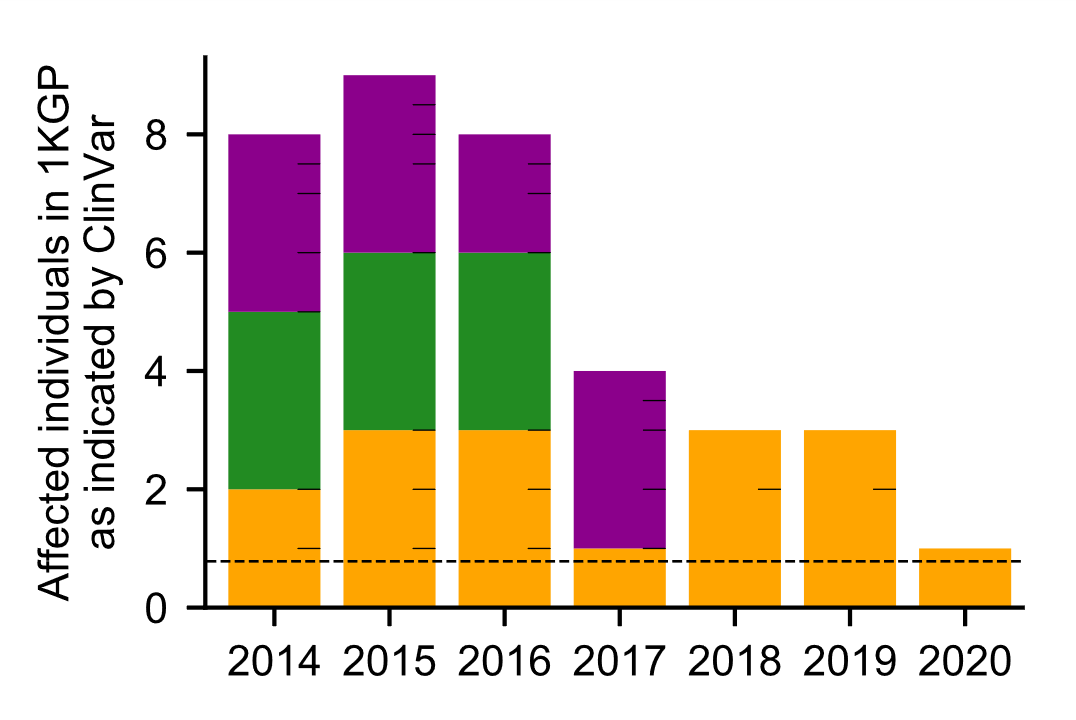
**

**B**

**Fig. S2.** (A) Indicated affected individuals in 1KGP when P variants with any number of stars are considered. (B) Indicated affected individuals in 1KGP when P or LP variants with any number of stars are considered (identical to Fig. 1D). The only difference is a single individual in 2017. Bar color, tick marks, and dashed lines are used as described in Fig. 1.


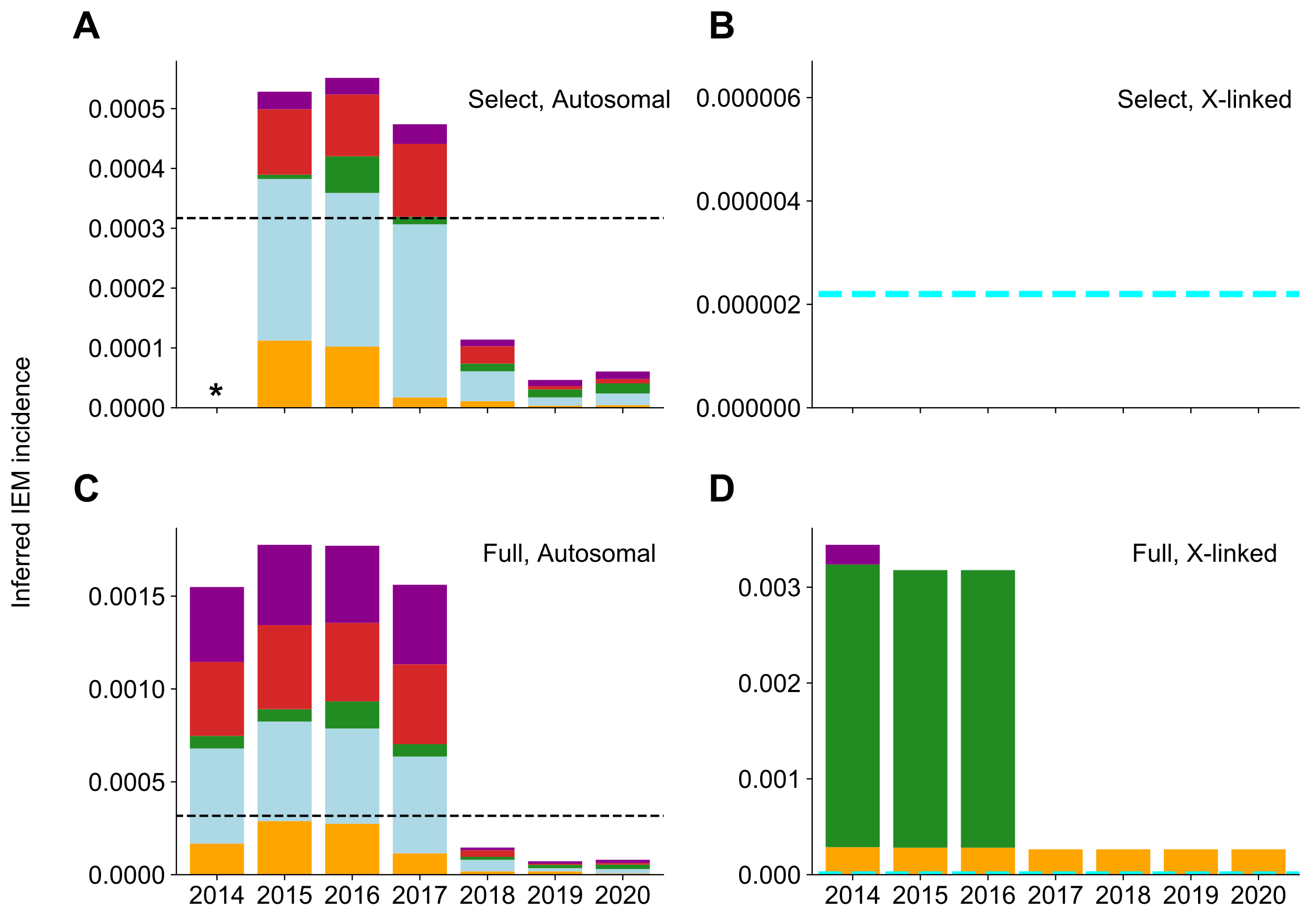


**Fig. S3.** Incidence of screened IEMs inferred by ClinVar variants. Dashed black line indicates expected incidence of autosomal screened IEMs of 1 in 3,200 births. Dashed cyan line indicates expected incidence of X-linked screened IEMs of 1 in 450,000 births. Bar colors are used as described in Fig. 1. The screened IEM incidence in 1KGP inferred by allele frequency of (A) Select autosomal ClinVar variants annotated as pathogenic. (B) Select X-linked ClinVar variants annotated as pathogenic. (C) Full autosomal ClinVar variants annotated as pathogenic. (D) Full X-linked ClinVar variants annotated as pathogenic. The 2015 BA1 guidelines were applied. When the 2018 BA1 guidelines are applied, the results did not change. *Data not available because existing review star framework was not in place until 2015.


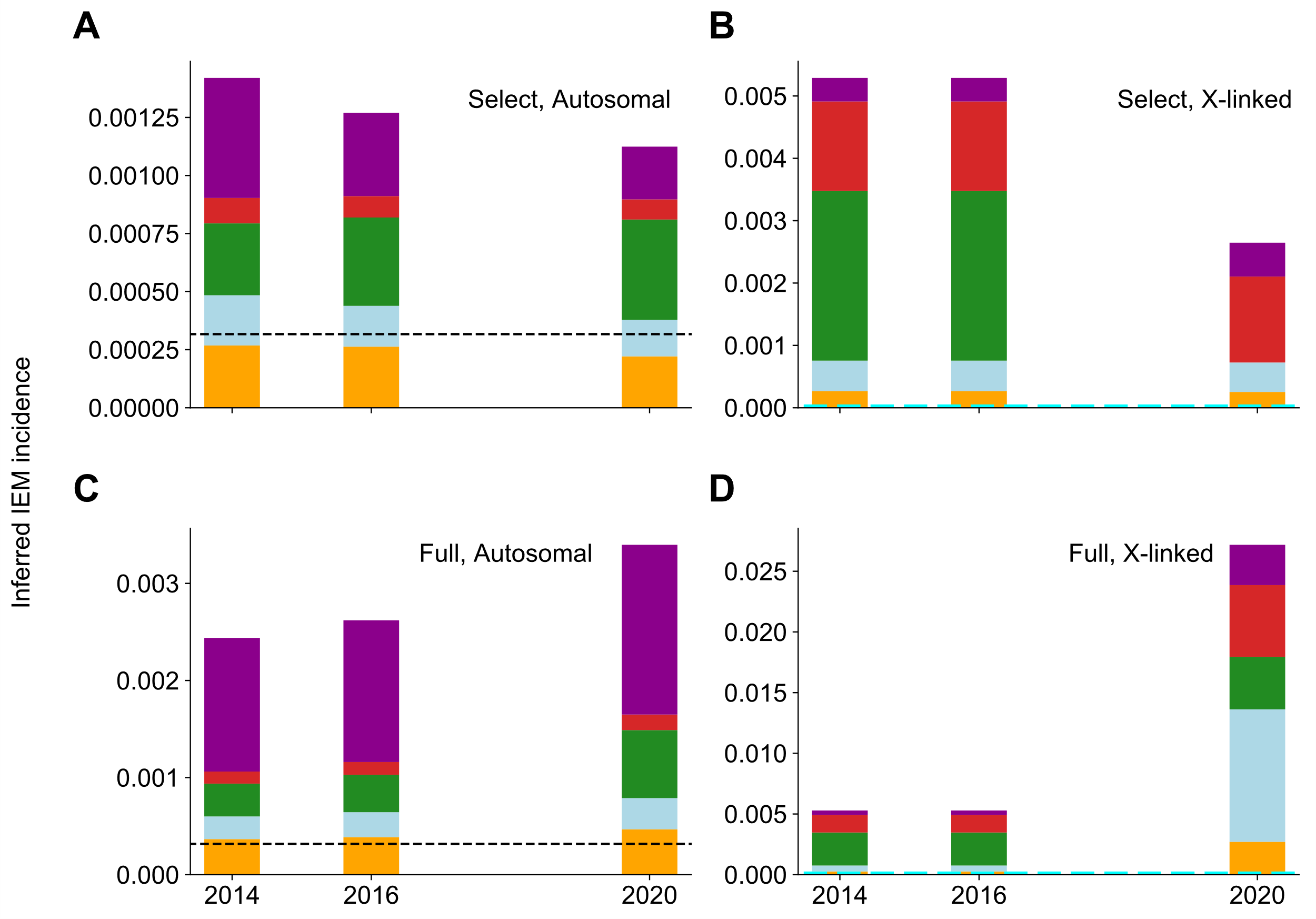


**Fig. S4.** Incidence of screened IEMs inferred by HGMD variants. Dashed black line indicates expected incidence of autosomal screened IEMs of 1 in 3,200 births. Dashed cyan line indicates expected incidence of X-linked screened IEMs of 1 in 450,000 births. Bar colors are used as described in Fig. 1. The screened IEM incidence in 1KGP inferred by allele frequency of (A) Select autosomal HGMD variants. (B) Select X-linked HGMD variants. (C) Full autosomal HGMD variants. (D) Full X-linked HGMD variants. The 2018 BA1 guidelines were applied.


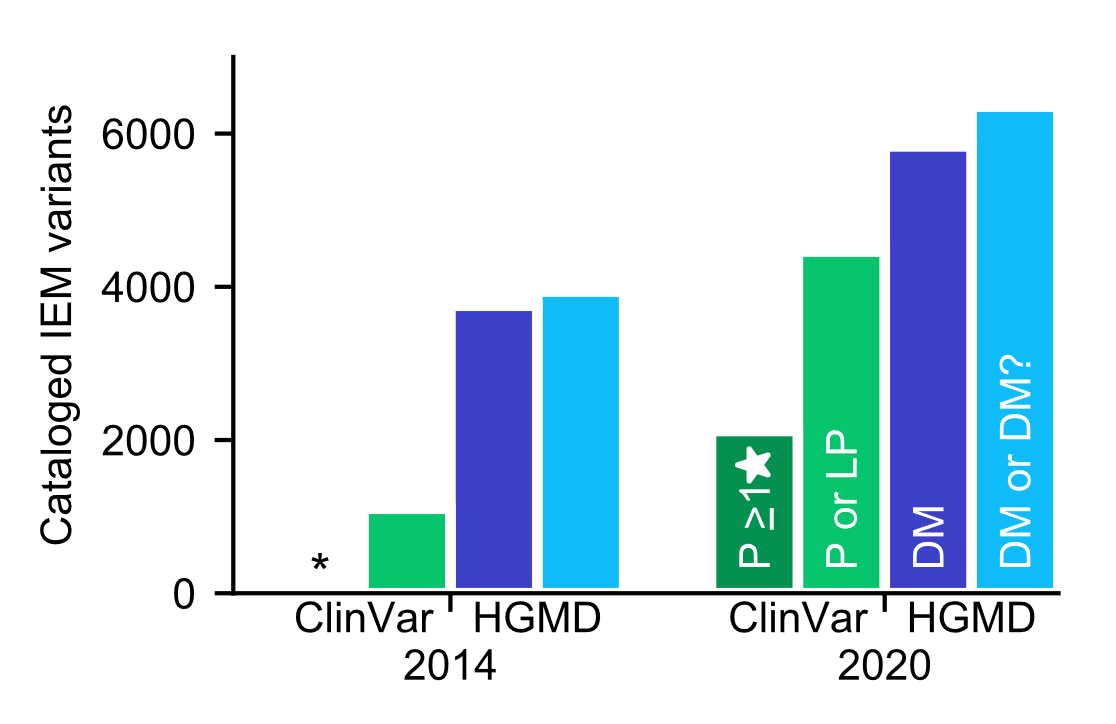

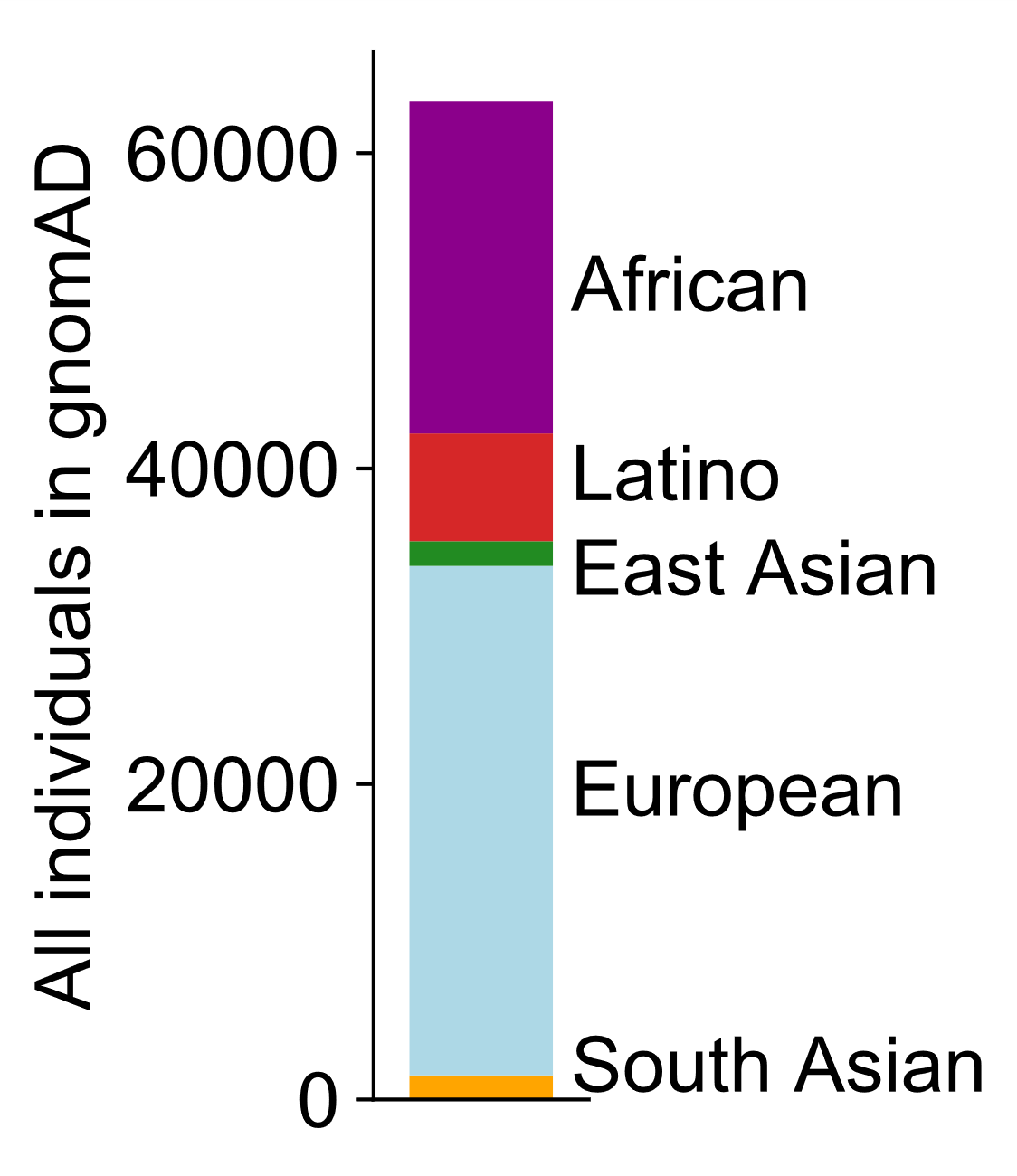

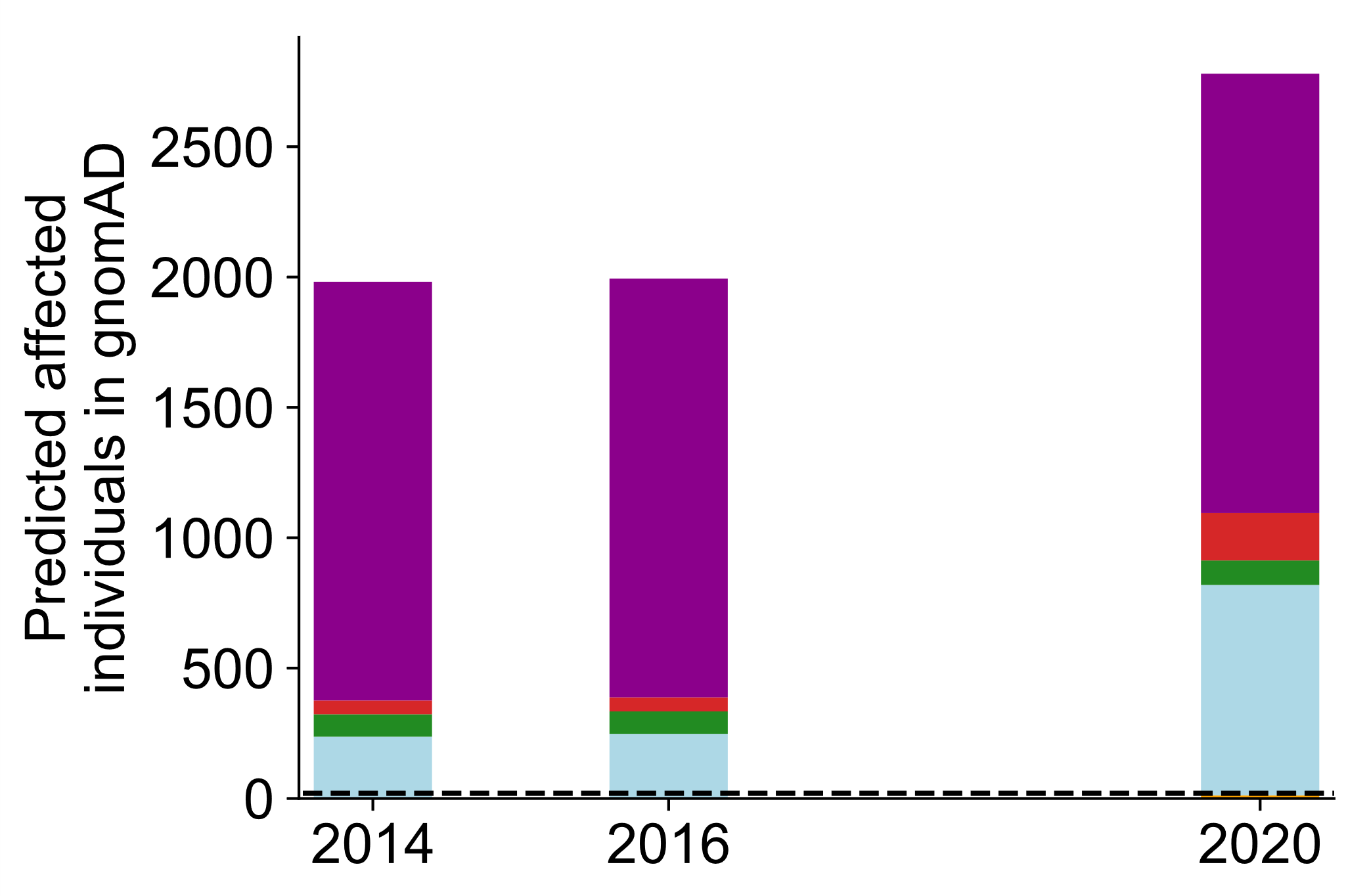

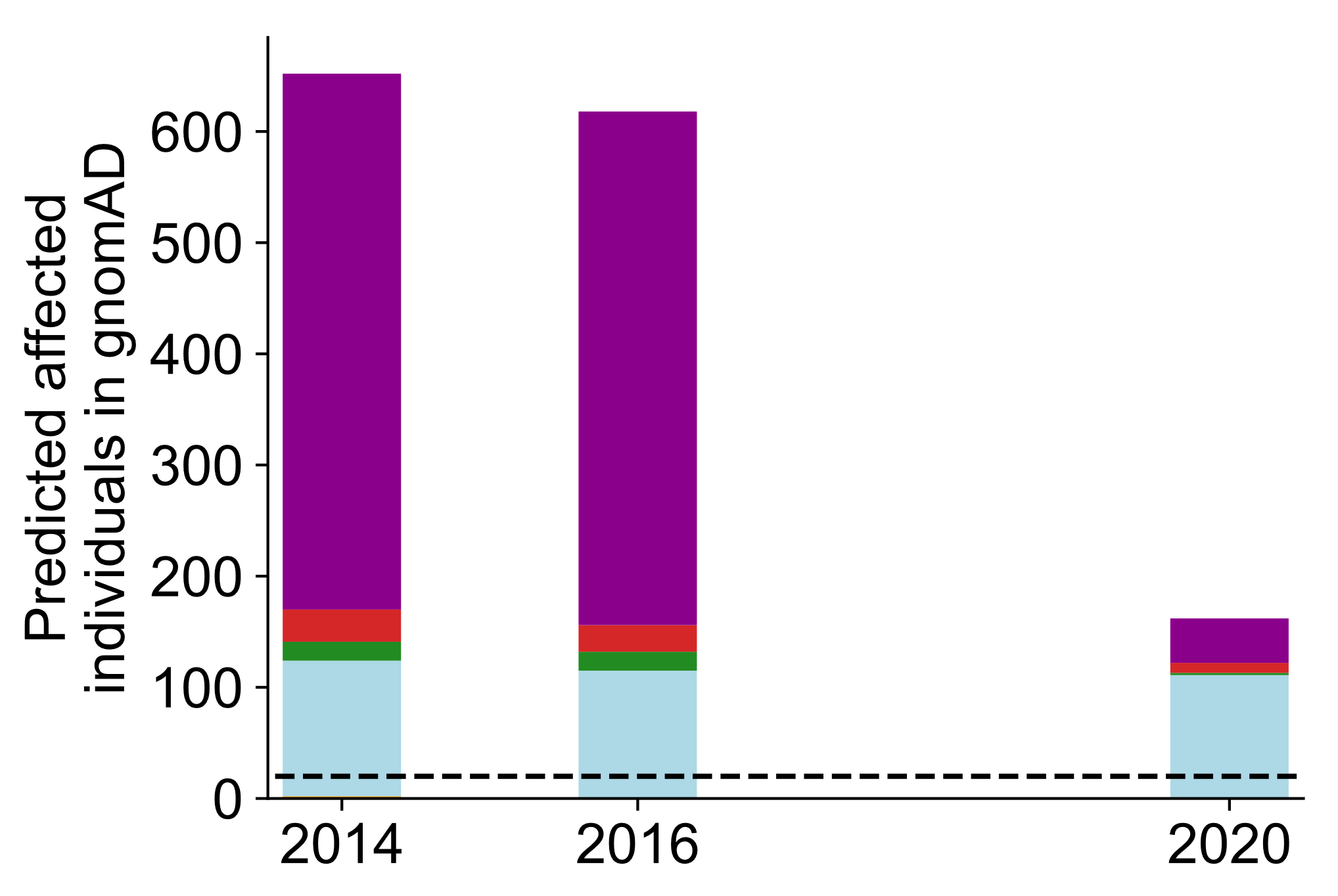

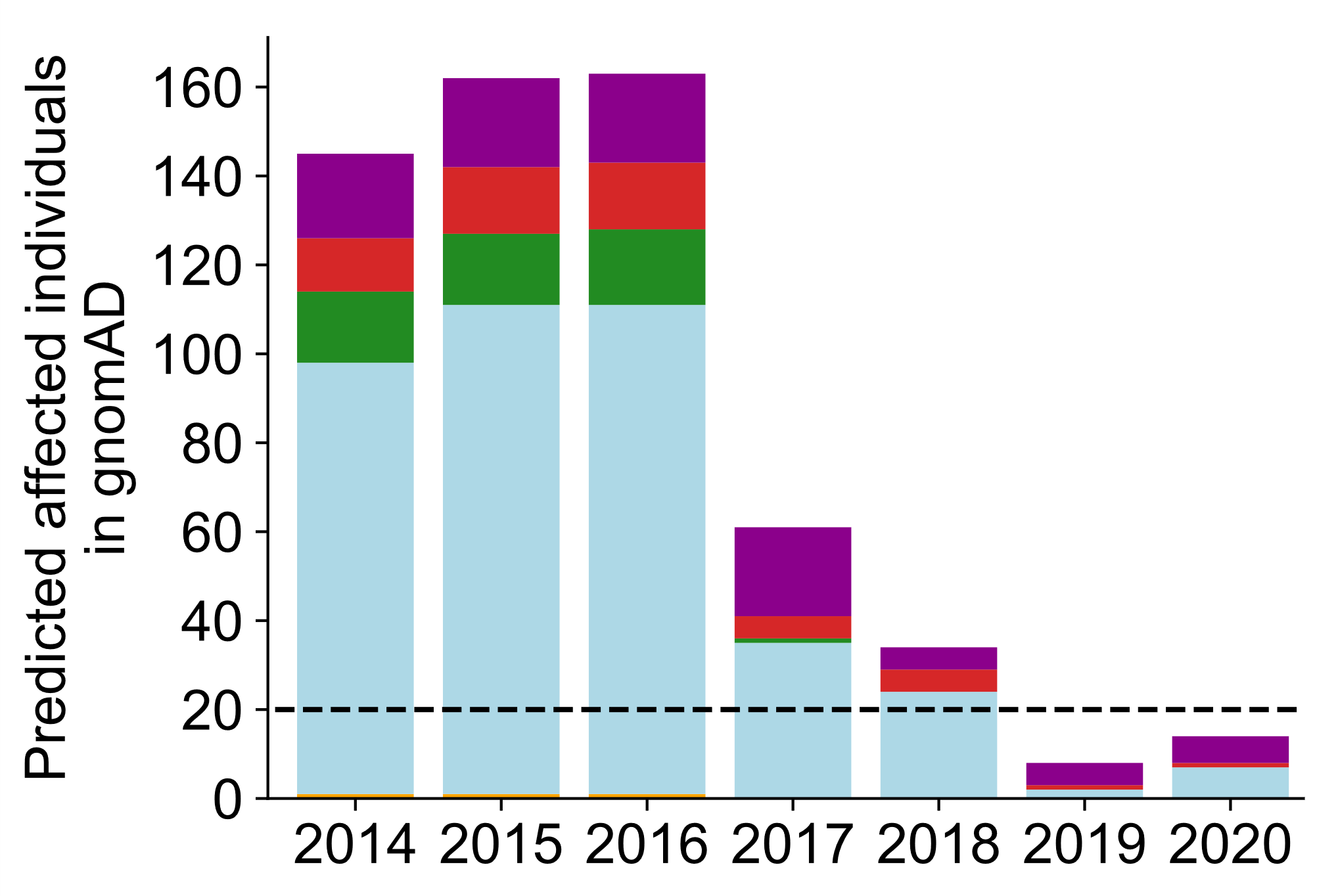

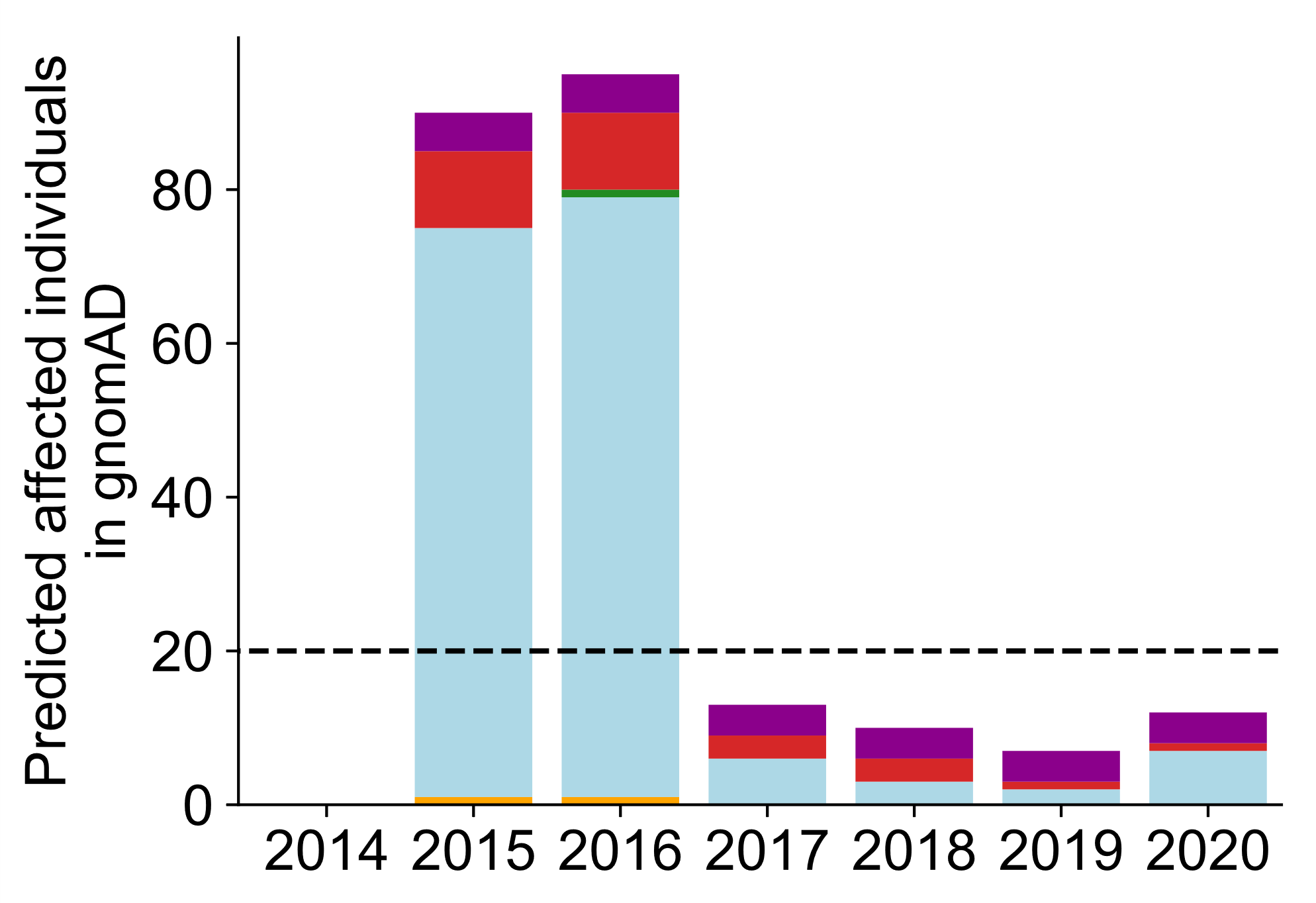


**B**

**A**

Full ClinVar

(P/LP & any stars)

Select ClinVar

(P & ≥1 stars)

Full HGMD

(DM or DM?)

Select HGMD

(DM)

Expected number of affected newborns

**C**

**D**

**E**

**F**

**G**

**
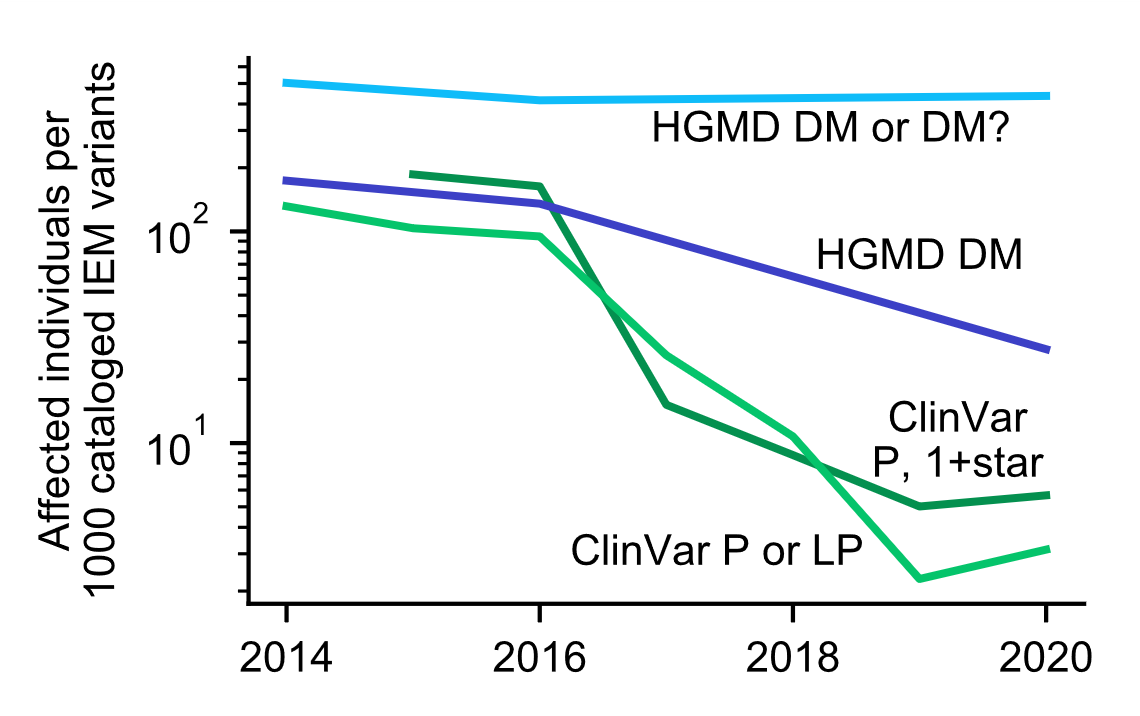
**

**Fig. S5.** Number of gnomAD individuals indicated affected for screened IEMs by ClinVar or HGMD over time. The 2015 BA1 guidelines were applied. (A) Number of screened IEM variants present in ClinVar or HGMD in 2014 and 2020 (identical to Fig. 1A). (B) Ancestry composition of individuals in gnomAD. Due to the imbalanced ancestry composition, ancestry skew is difficult to discern visually in the following panels. (C-F) Bars are colored by ancestry as shown in (B). Dashed black lines indicate the aggregate population incidence of screened IEMs. The number of gnomAD individuals with a pathogenic genotype for a variant in (C) Select ClinVar variants annotated as pathogenic, defined as variants with a P interpretation with at least 1 review star. Variants that also have conflicting interpretations (with VUS or B/LB) with 1 or more review stars are removed. (D) Full ClinVar variants annotated as pathogenic, defined as variants with a P or LP interpretation. Variants that also have conflicting interpretations (with VUS or B/LB) are removed. (E) Select HGMD variants, defined as variants classified as DM. 2014, 2016, and 2020 are shown because they are the years for which we have archived HGMD data. (F) Full HGMD variants, defined as variants classified as DM or DM?. (G) The number of affected individuals relative to the number of variants classified in each variant set. *Data not available because existing review star framework was not in place until 2015.

**
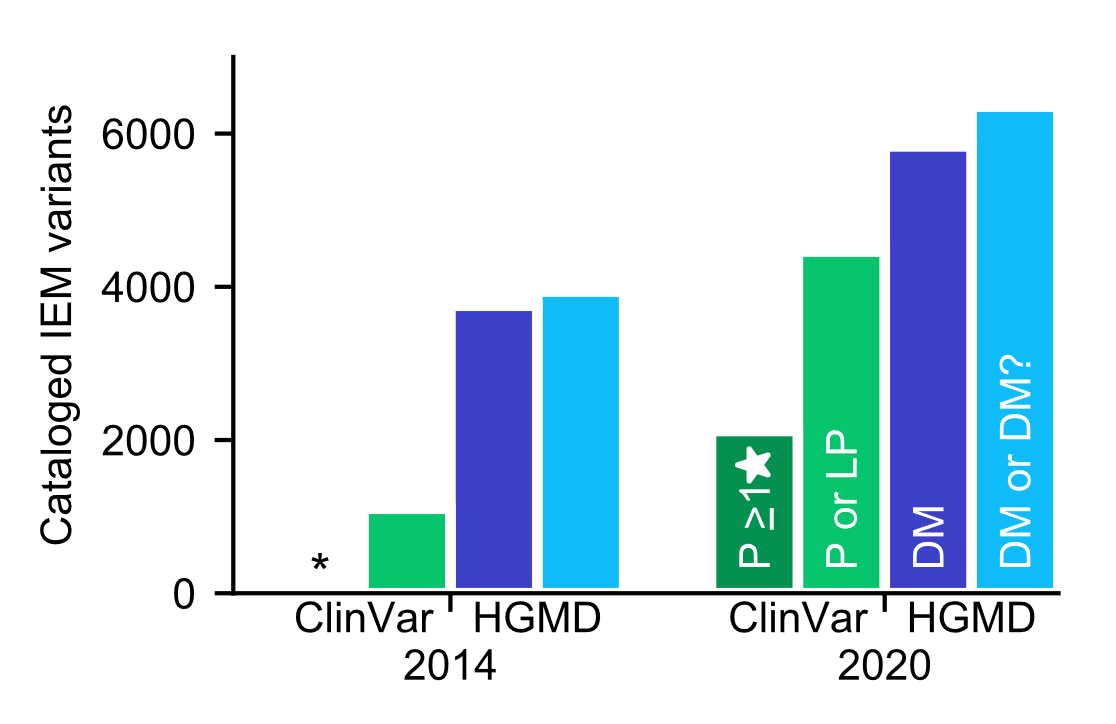

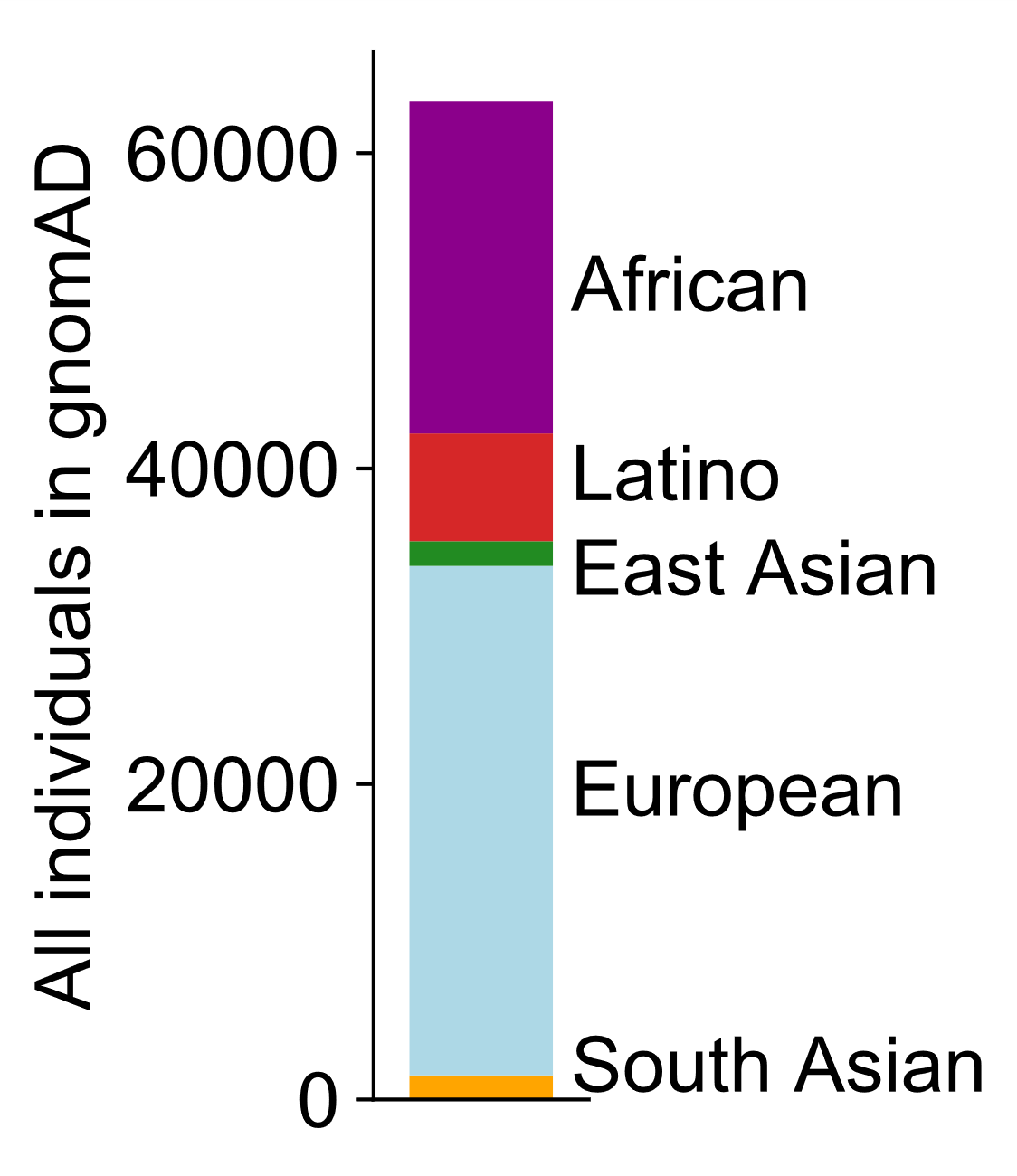

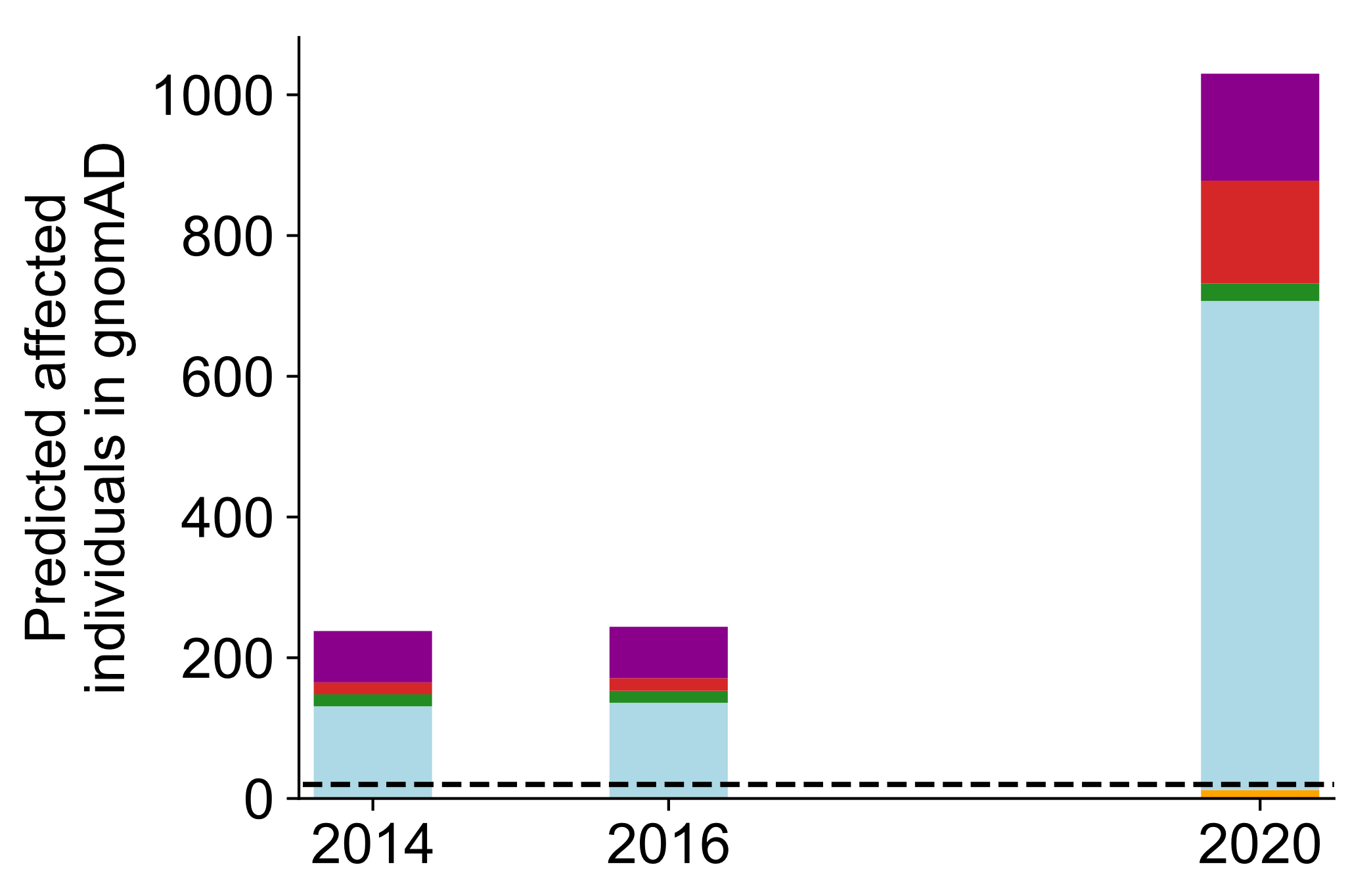

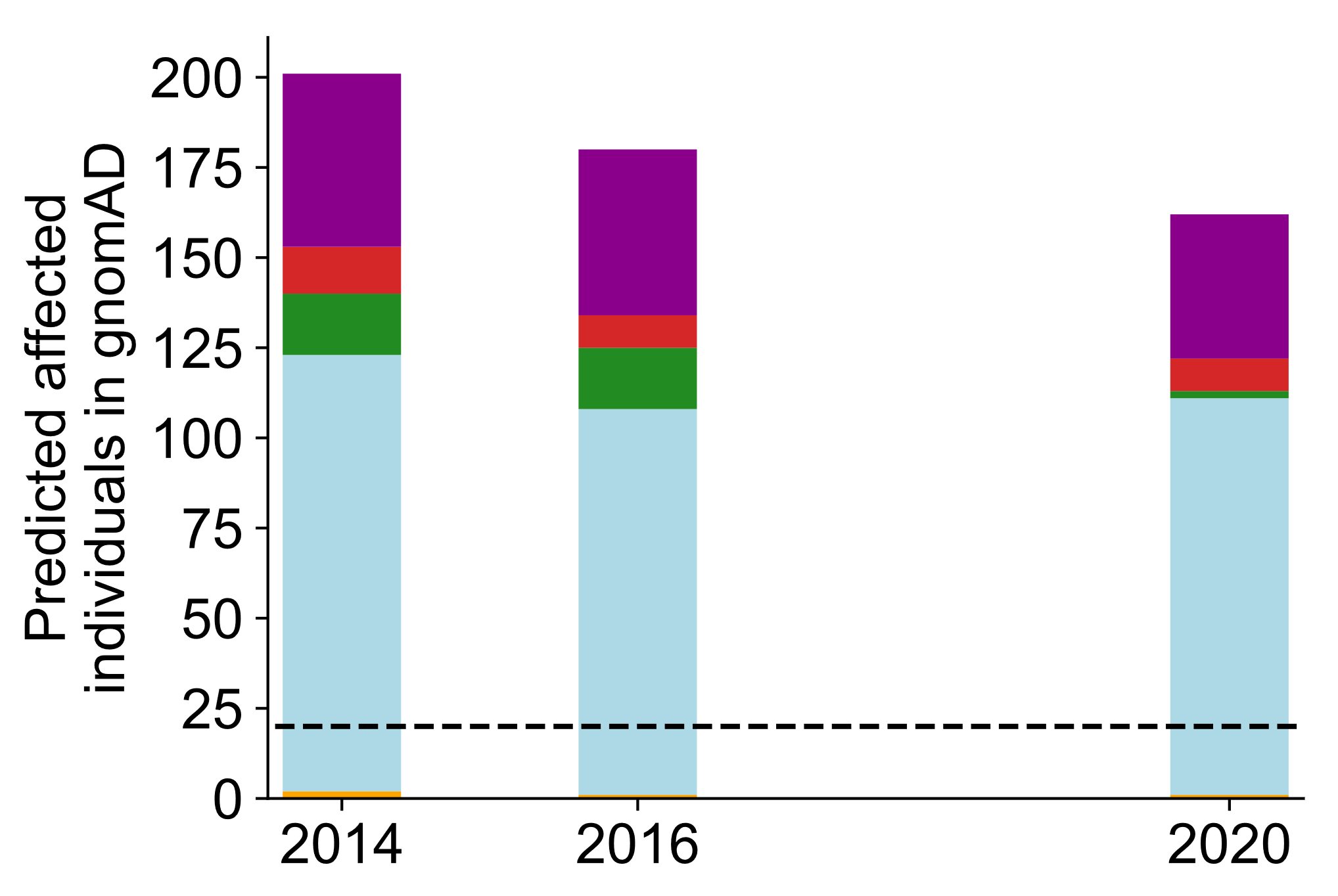

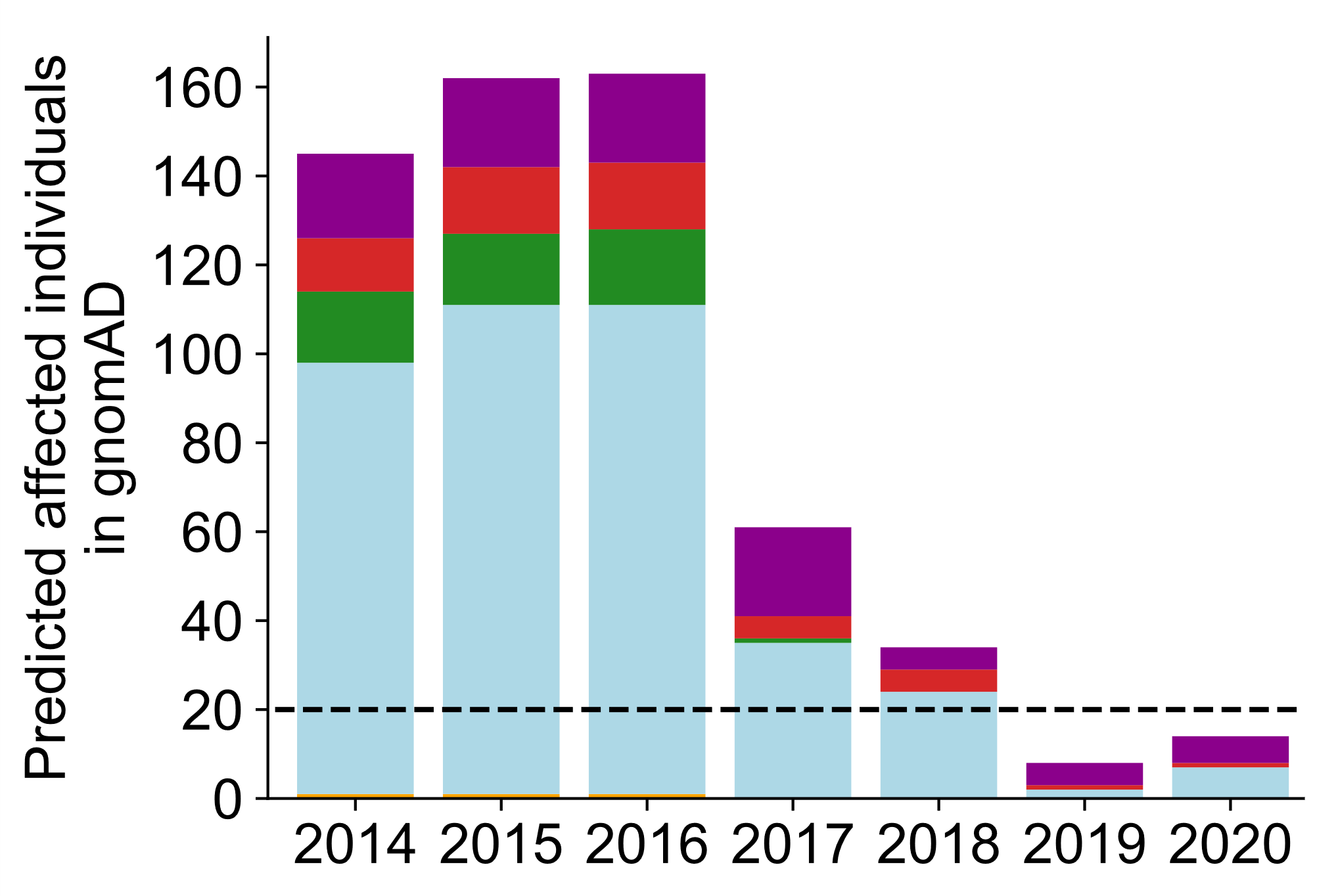

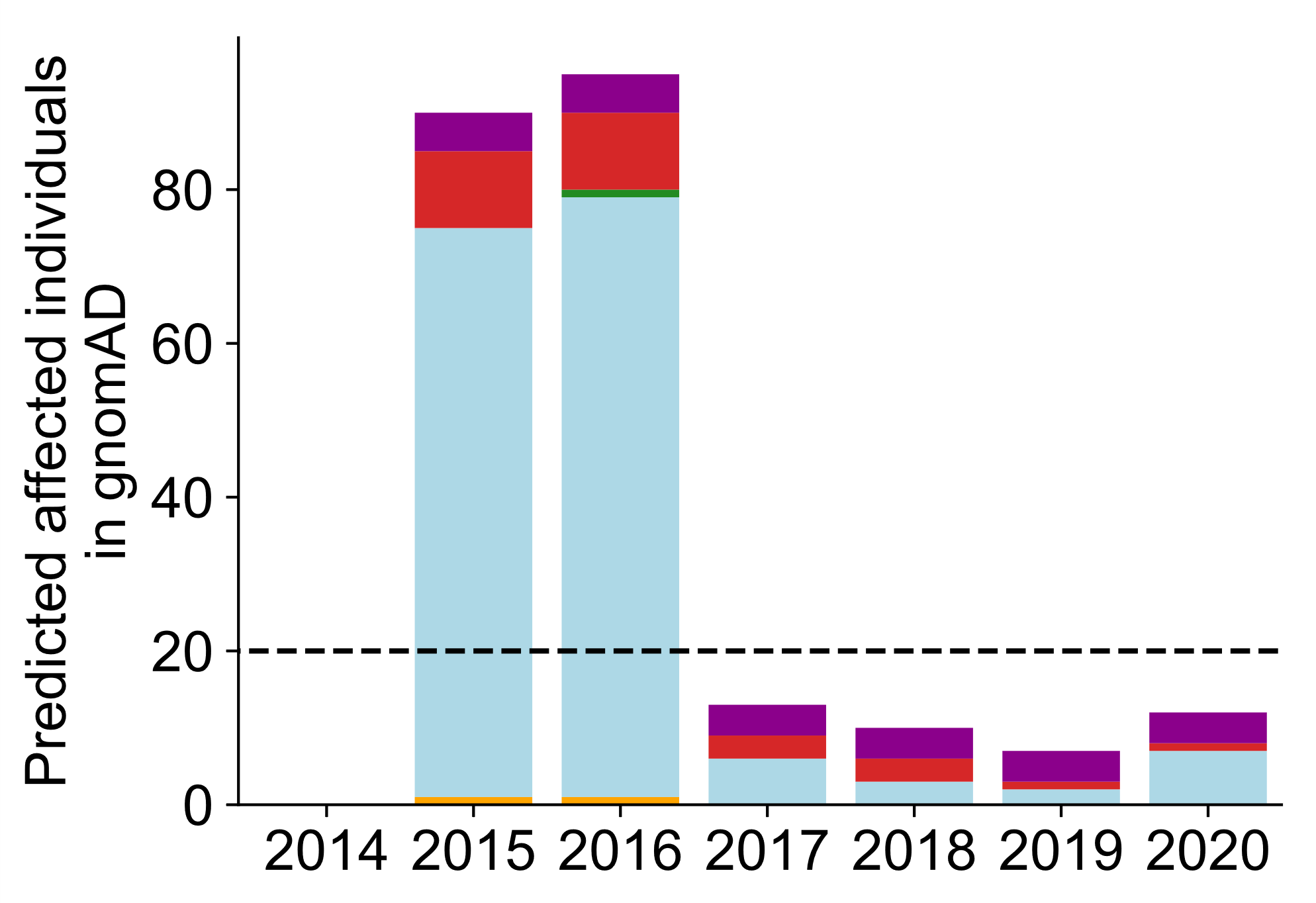
**

**B**

**A**

Full ClinVar

(P/LP & any stars)

Select ClinVar

(P & ≥1 stars)

Full HGMD

(DM or DM?)

Select HGMD

(DM)

Expected number of affected newborns

**D**

**F**


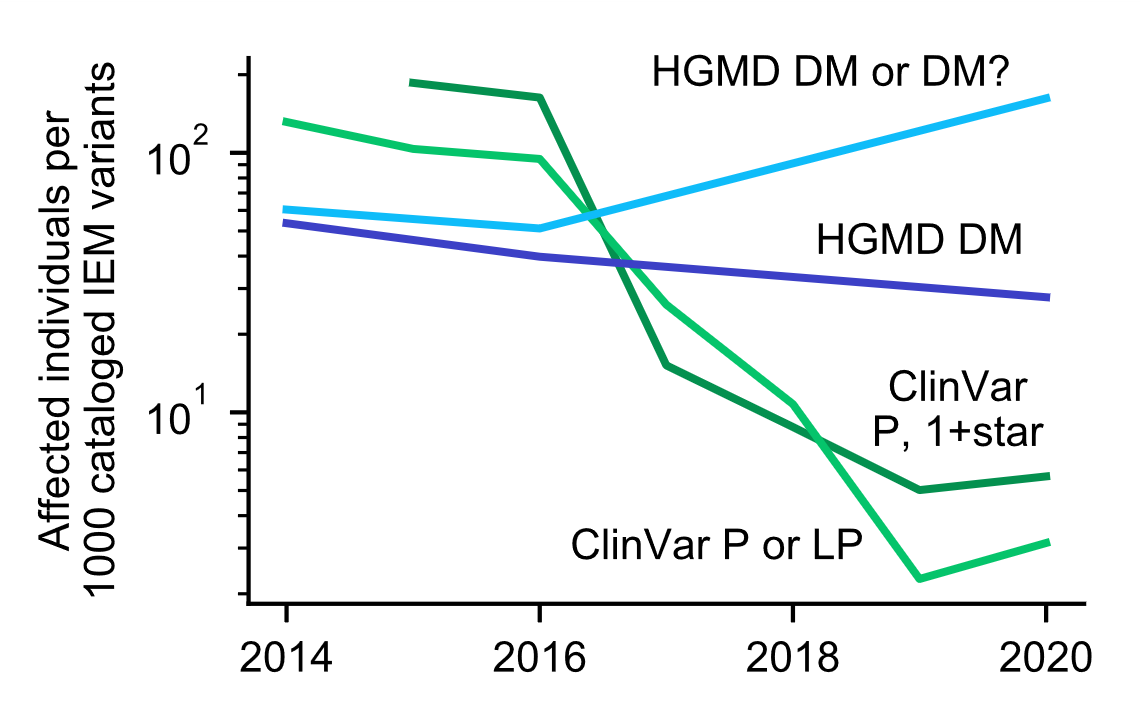


**E**

**C**

**G**

**Fig. S6.** Number of gnomAD individuals indicated affected for screened IEMs by ClinVar or HGMD over time. The 2018 BA1 guidelines were applied. (A) Number of screened IEM variants present in ClinVar or HGMD in 2014 and 2020 (identical to Fig. 1A). (B) Ancestry composition of individuals in gnomAD. Due to the imbalanced ancestry composition, ancestry skew is difficult to discern visually in the following panels. (C-F) Bars are colored by ancestry as shown in (B). Dashed black lines indicate the aggregate population incidence of screened IEMs. The number of gnomAD individuals with a pathogenic genotype for a variant in (C) Select ClinVar variants annotated as pathogenic, defined as variants with a P interpretation with at least 1 review star. Variants that also have conflicting interpretations (with VUS or B/LB) with 1 or more review stars are removed. (D) Full ClinVar variants annotated as pathogenic, defined as variants with a P or LP interpretation. Variants that also have conflicting interpretations (with VUS or B/LB) are removed. (E) Select HGMD variants, defined as variants classified as DM. 2014, 2016, and 2020 are shown because they are the years for which we have archived HGMD data. (F) Full HGMD variants, defined as variants classified as DM or DM?. (G) The number of affected individuals relative to the number of variants classified in each variant set. *Data not available because existing review star framework was not in place until 2015.


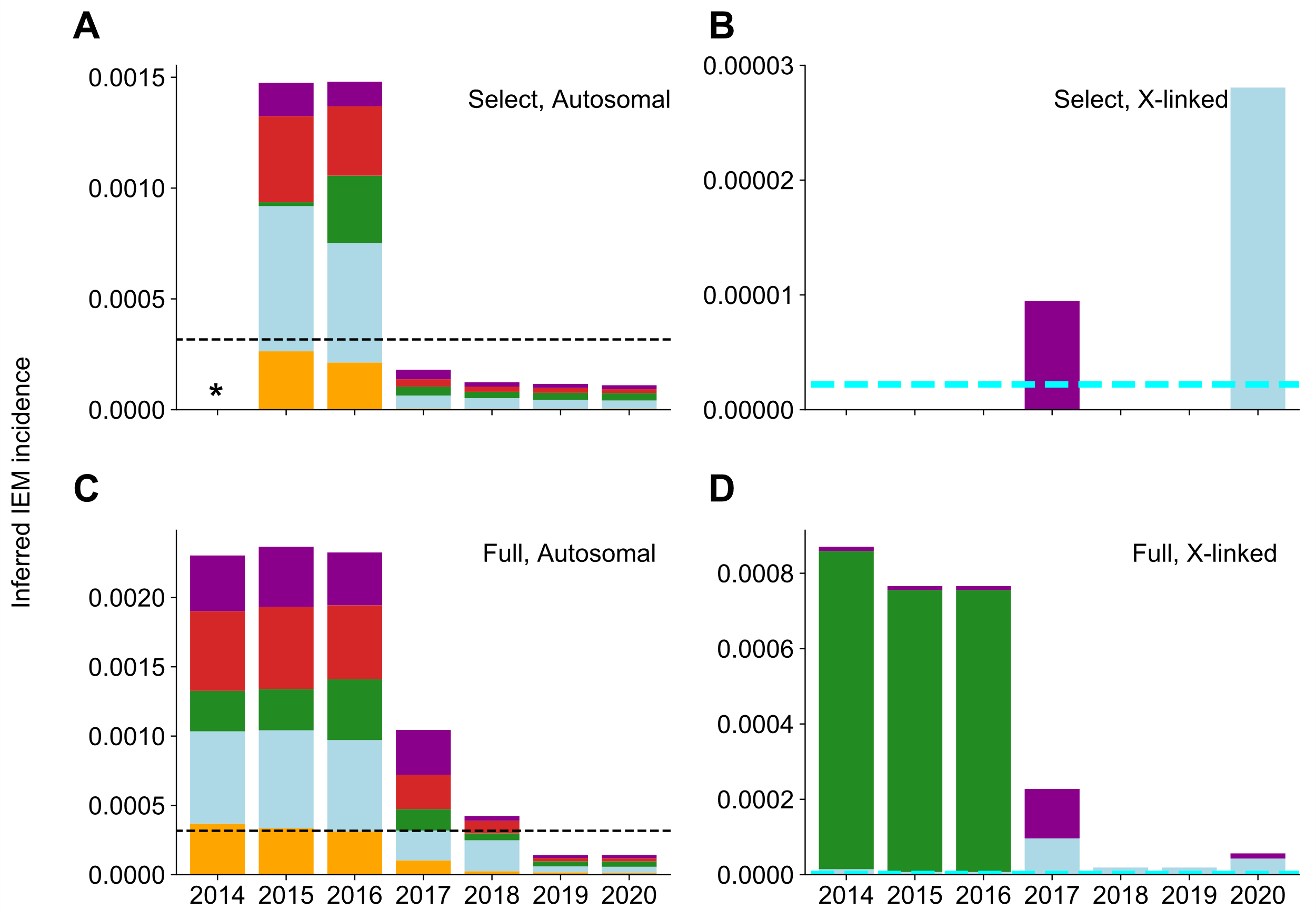


**Fig. S7.** Incidence of screened IEMs inferred from ClinVar variants and gnomAD allele frequencies. The 2015 BA1 guidelines were applied. When the 2018 BA1 guidelines are applied, the results did not change. Dashed black line indicates expected incidence of autosomal screened IEMs of 1 in 3,200 births. Dashed cyan line indicates expected incidence of X-linked screened IEMs of 1 in 450,000 births. Bar colors are used as described in Fig. 1. The screened IEM incidence in gnomAD inferred by allele frequency of (A) Select autosomal ClinVar variants annotated as pathogenic. (B) Select X-linked ClinVar variants annotated as pathogenic. (C) Full autosomal ClinVar variants annotated as pathogenic. (D) Full X-linked ClinVar variants annotated as pathogenic. *Data not available because existing review star framework was not in place until 2015.


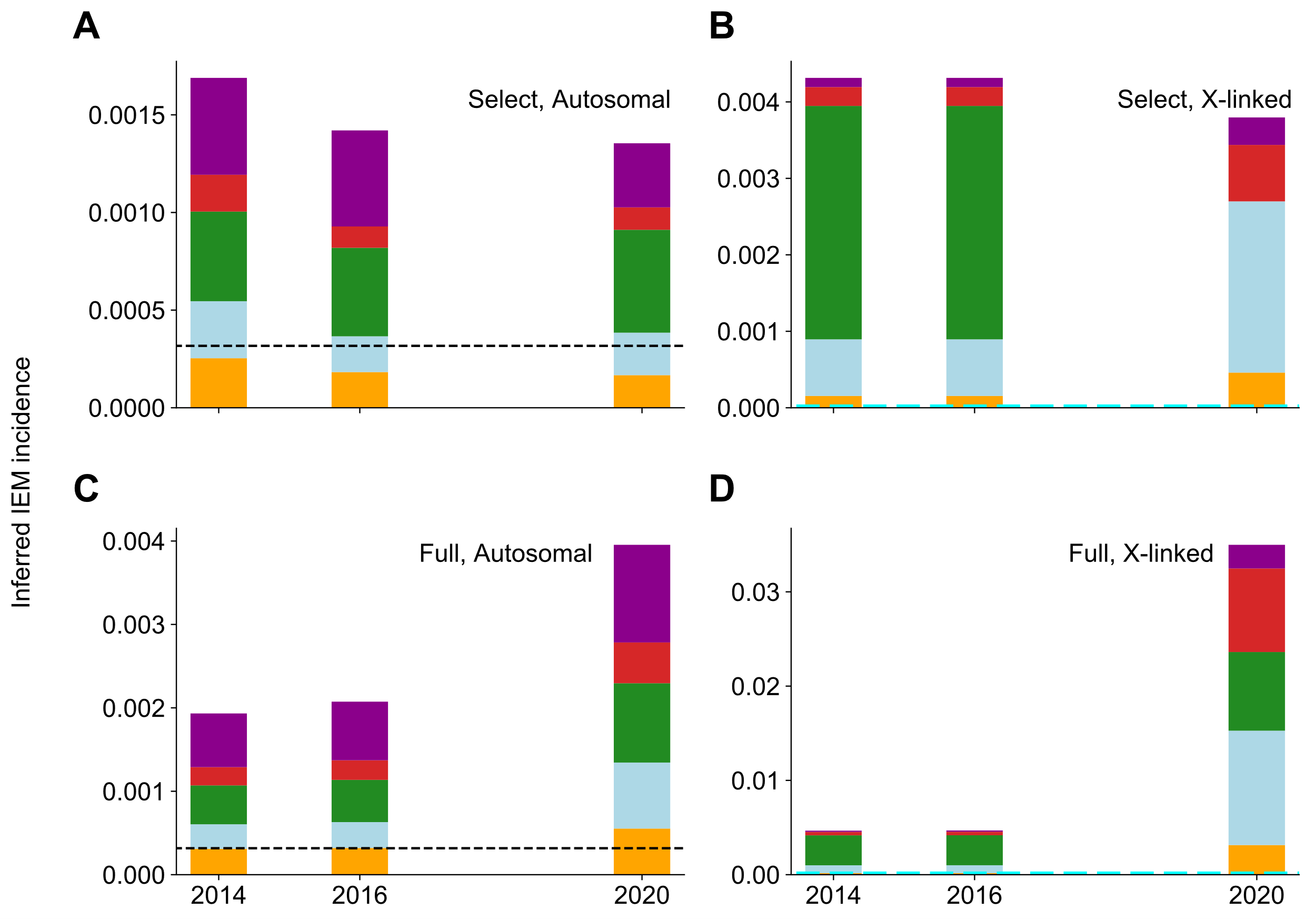


**Fig. S8.** Incidence of screened IEMs inferred by HGMD variants and gnomAD allele frequencies. The 2018 BA1 guidelines were applied. Dashed black line indicates expected incidence of autosomal screened IEMs of 1 in 3,200 births. Dashed cyan line indicates expected incidence of X-linked screened IEMs of 1 in 450,000 births. Bar colors are used as described in Fig. 1. The screened IEM incidence in 1KGP inferred by allele frequency of (A) Select autosomal HGMD variants. (B) Select X-linked HGMD variants. (C) Full autosomal HGMD variants. (D) Full X-linked HGMD variants.


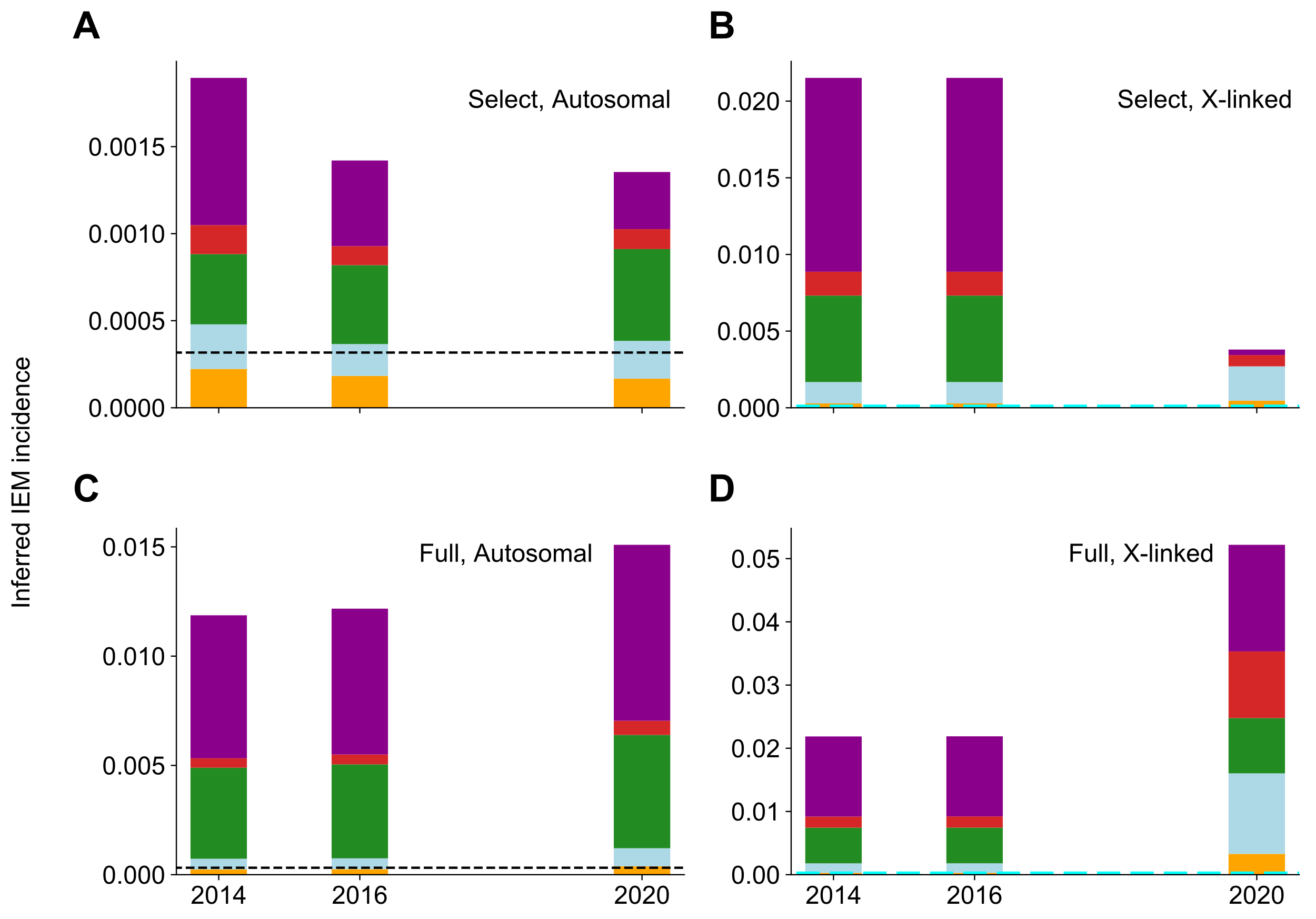


**Fig. S9.** Incidence of screened IEMs inferred by HGMD variants. The 2015 BA1 guidelines were applied. Dashed black line indicates expected incidence of autosomal screened IEMs of 1 in 3,200 births. Dashed cyan line indicates expected incidence of X-linked screened IEMs of 1 in 450,000 births. Bar colors are used as described in Fig. 1. The screened IEM incidence in 1KGP inferred by allele frequency of (A) Select autosomal HGMD variants. (B) Select X-linked HGMD variants. (C) Full autosomal HGMD variants. (D) Full X-linked HGMD variants.

**
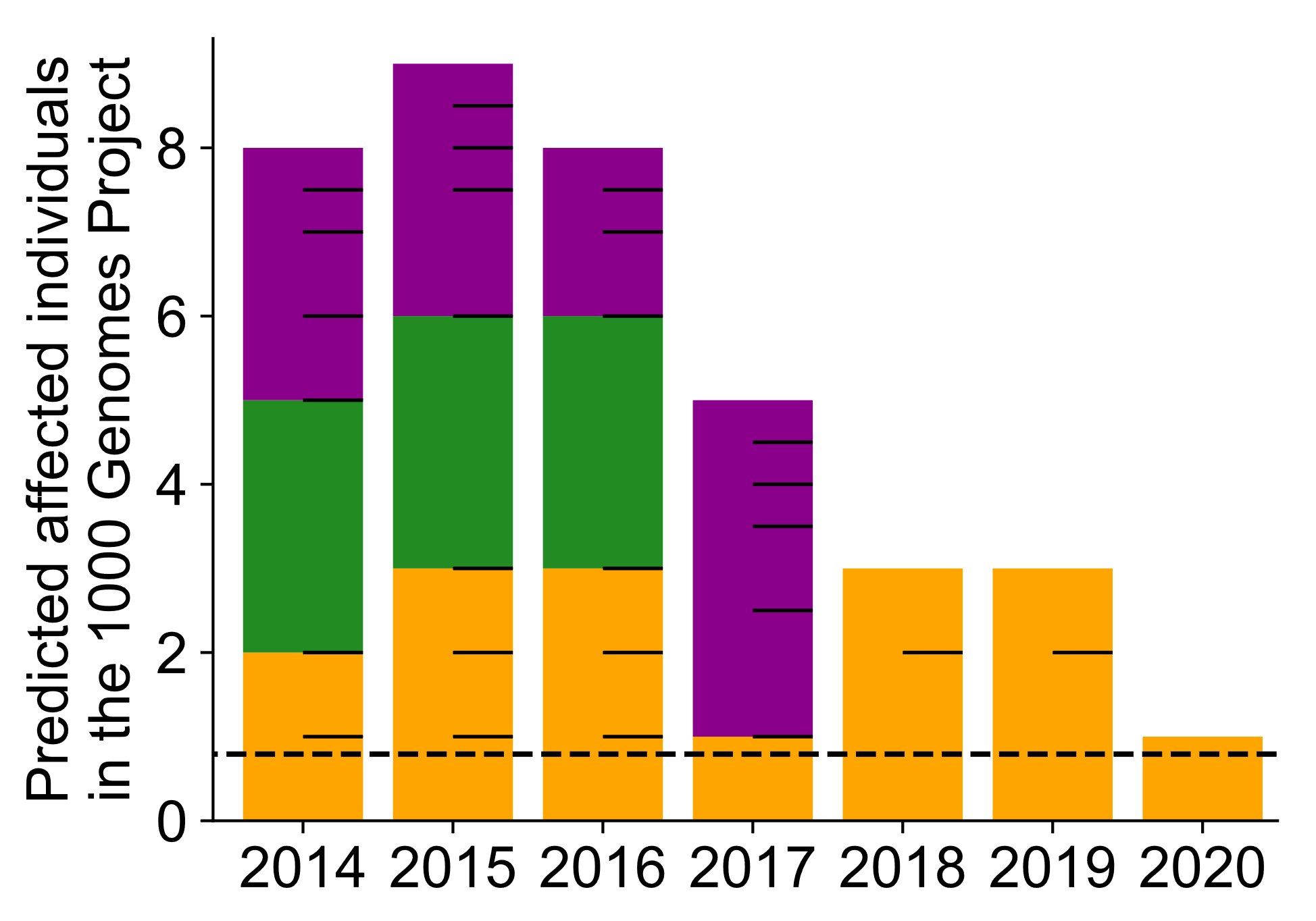

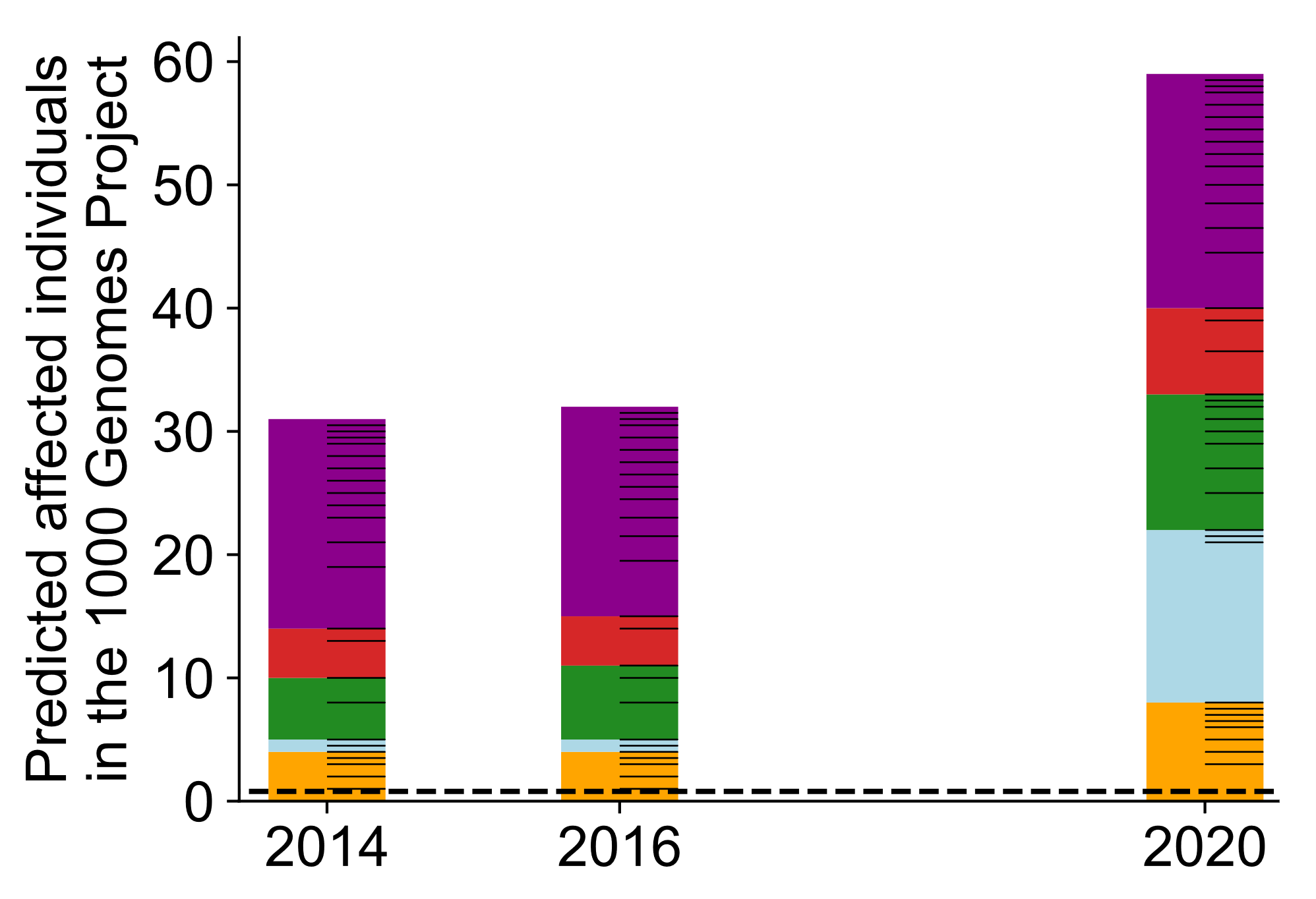

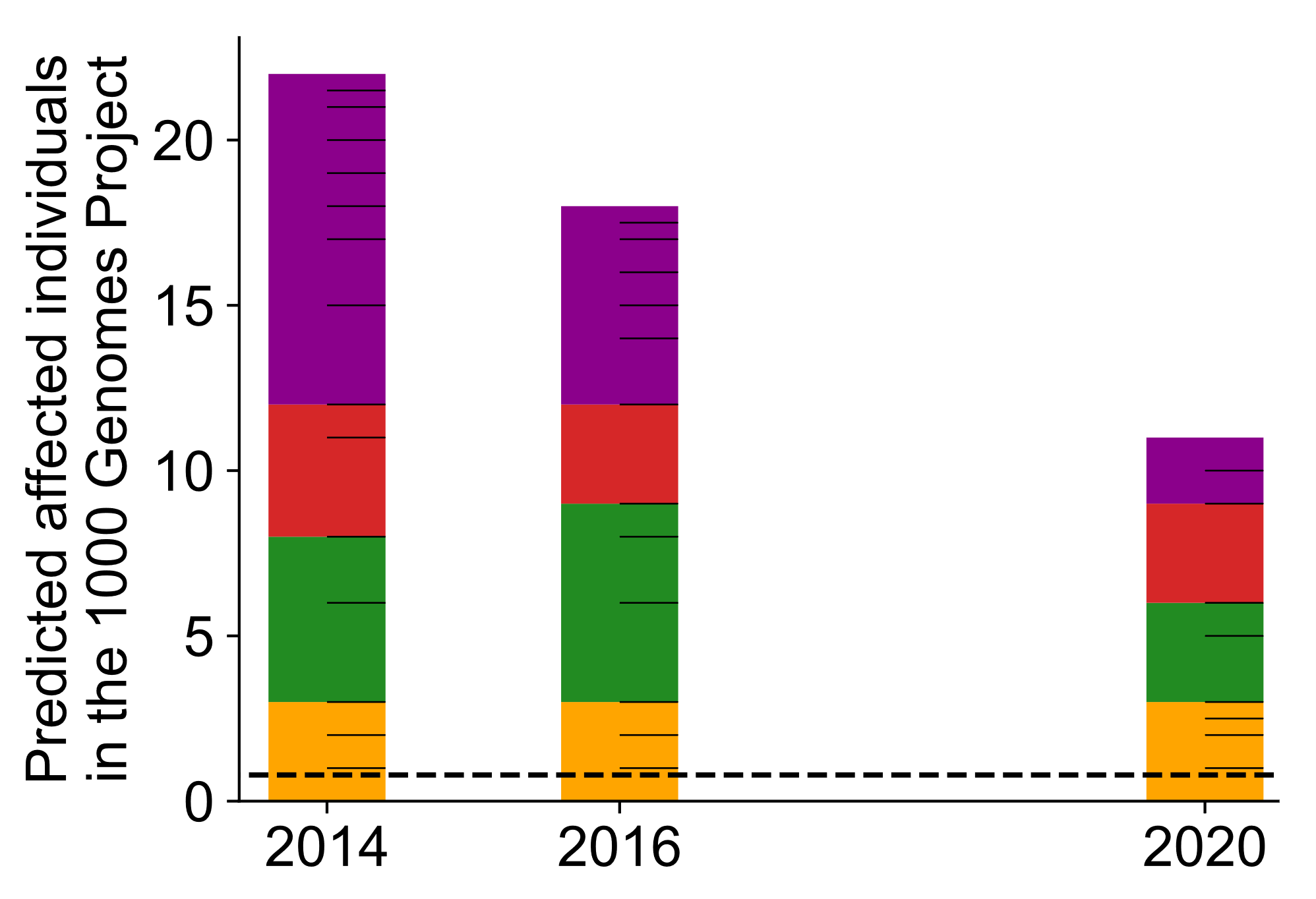

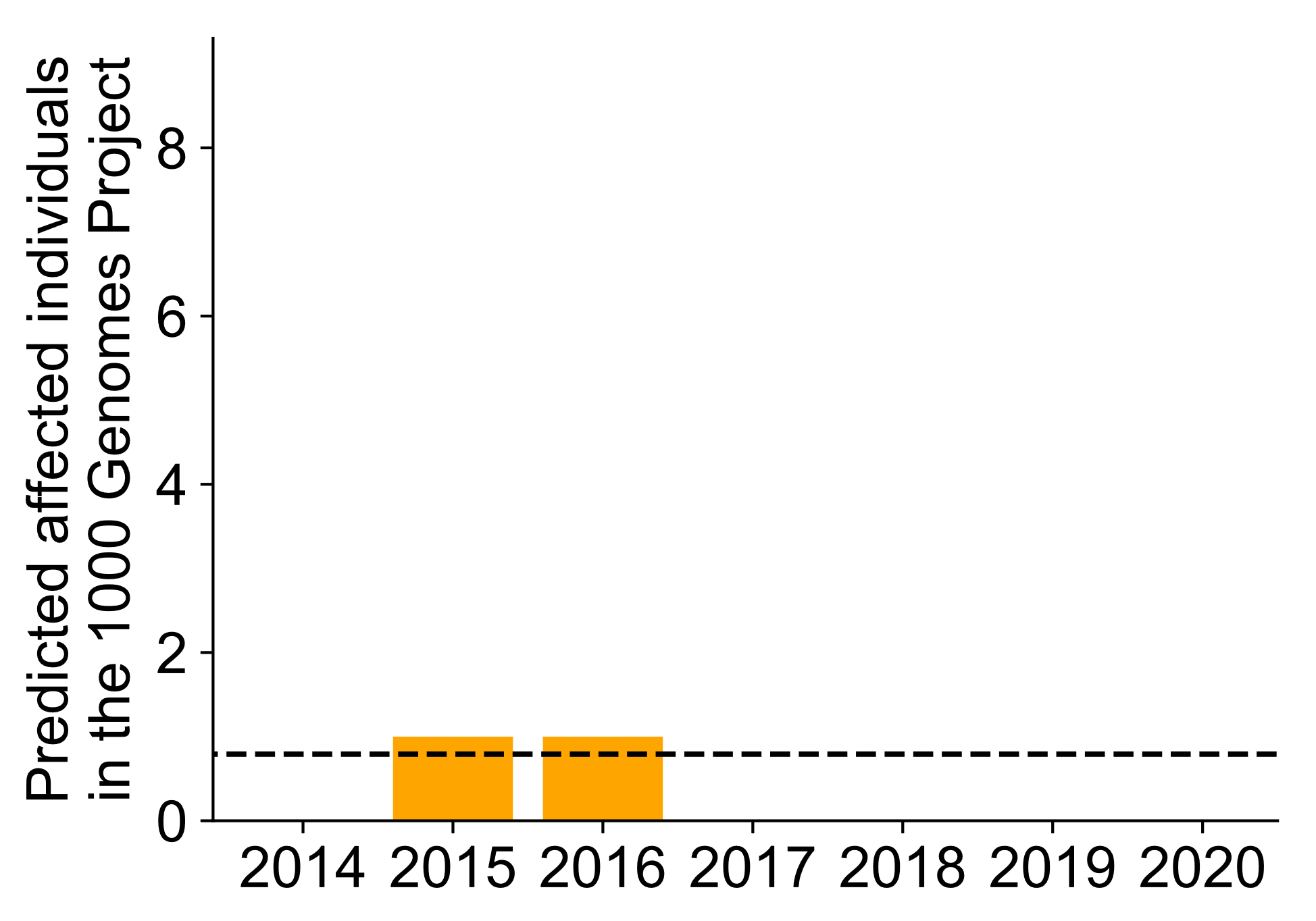
**

**D**

**C**

**B**

**A**

**
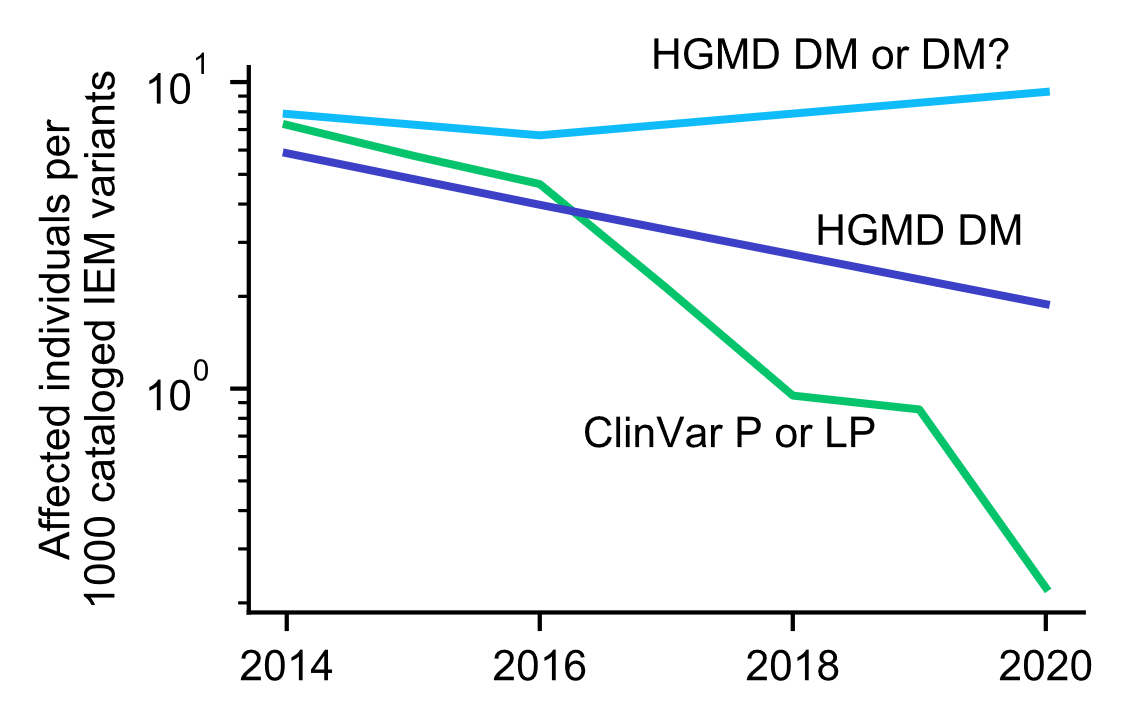
**

**E**

**Fig. S10.** Number of 1KGP individuals indicated affected for screened IEMs with the 2018 BA1 guidelines implemented. Bar coloring, tick marks, and dashed lines are used as described in Fig. 1. The number of 1KGP individuals with an implied pathogenic genotype for a variant in (A) Select ClinVar variants annotated as pathogenic. (B) Full ClinVar variants annotated as pathogenic. (C) Select HGMD variants. (D) Full HGMD variants. (E) The number of affected individuals relative to the number of variants classified in each variant set.


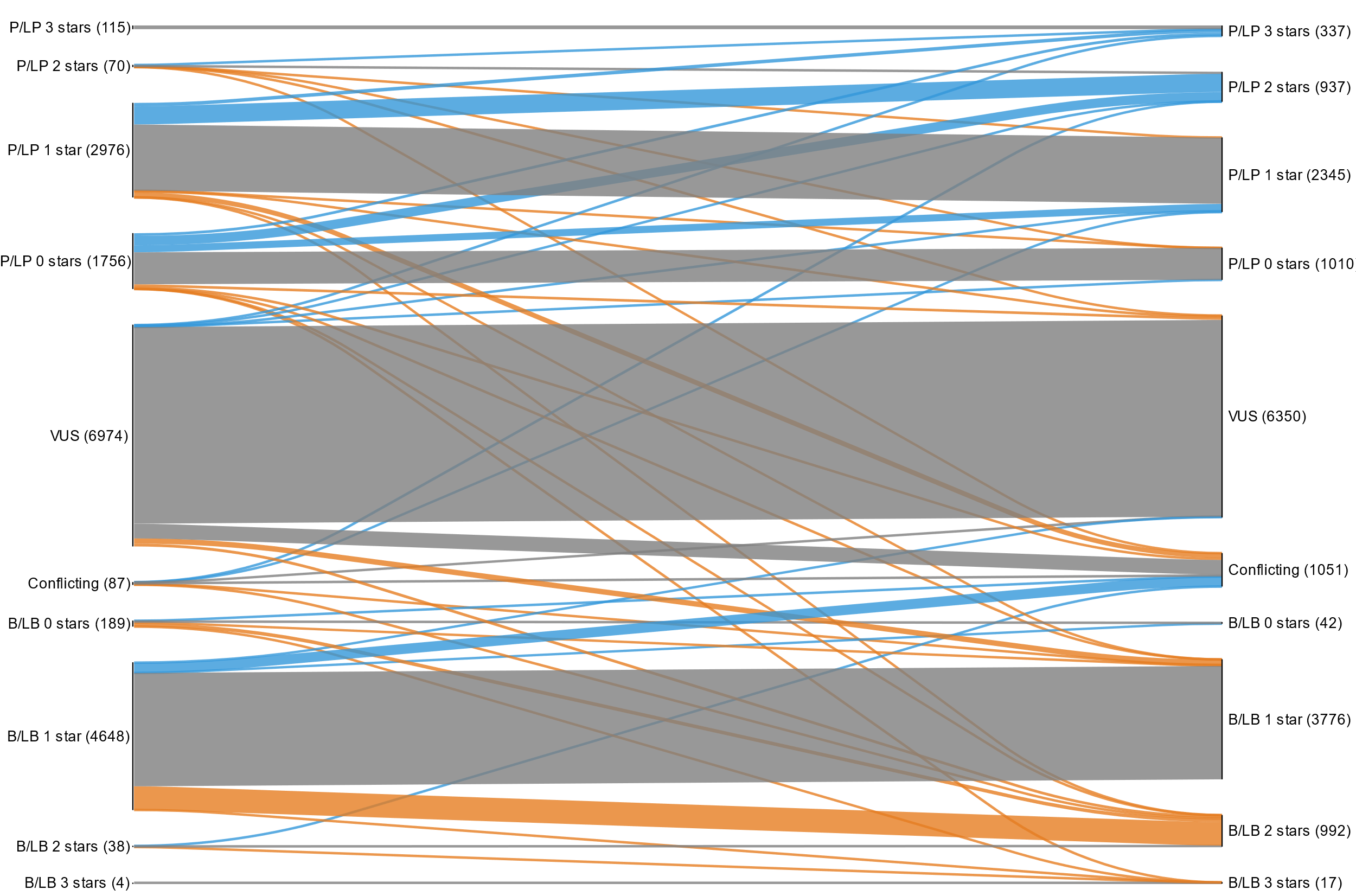


**Fig. S11.** Reclassification paths of all screened IEM ClinVar variants from 2014 (or first submission thereafter) to 2020, visualized in a Sankey plot in which line width represents the number of reclassified variants. Blue lines indicate increasing annotated pathogenicity or review stars, orange lines indicate increasing annotated benignity or reduced confidence of pathogenicity, and gray lines indicate no change. Numbers in parentheses provide variant counts of initial and final classifications for each category.

**
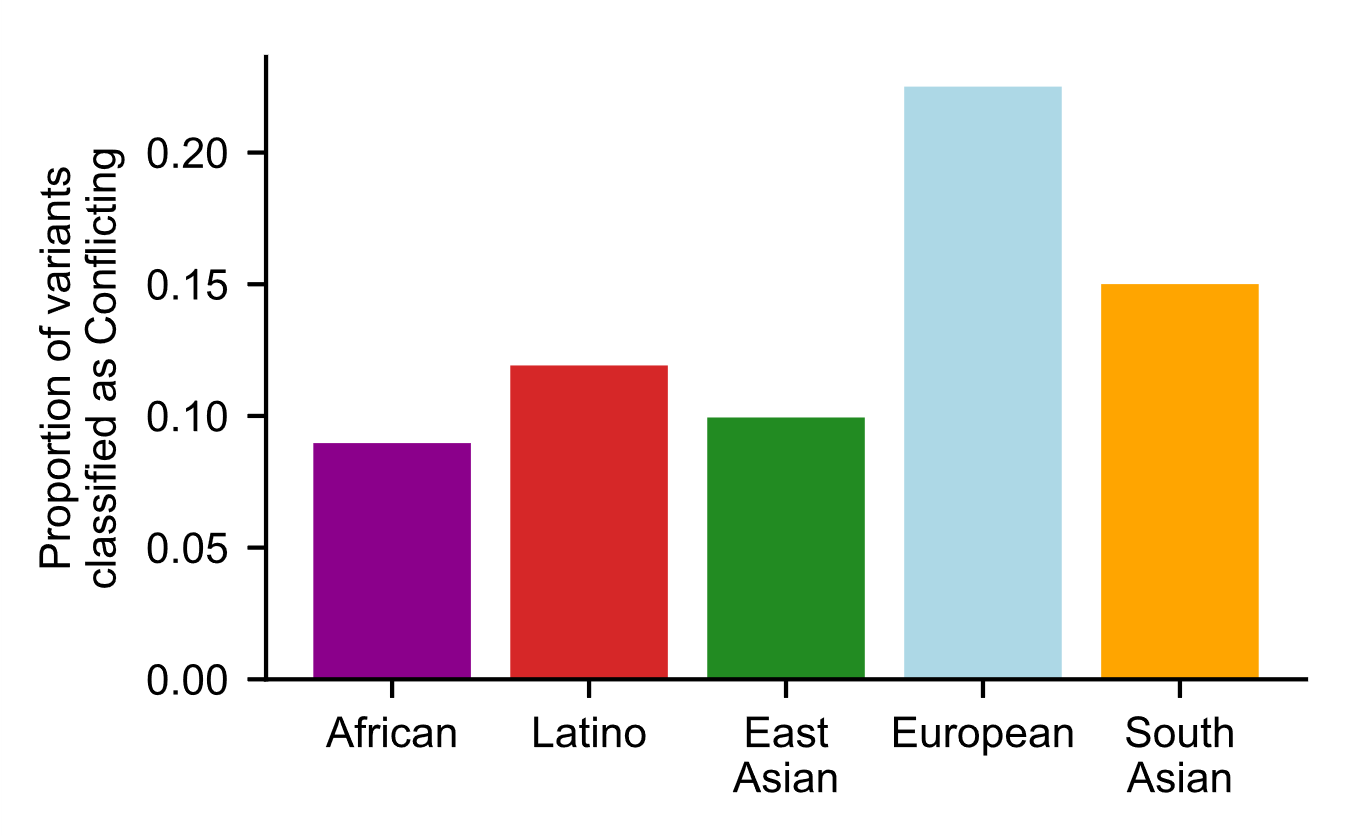
**

**Fig. S12.** For each ancestry, proportion of ClinVar variants that are classified as Conflicting. For each variant, we used gnomAD exomes to identify the ancestry group with the highest MAF, and we assigned the variant to that ancestry group.
