# Supplementary material for: ClinVar and HGMD genomic variant classification accuracy has improved over time, as measured by implied disease burden
Source: Genome Med. 2023 Jul 13;15:51. doi: 10.1186/s13073-023-01199-y (PMC10347827; doi:10.1186/s13073-023-01199-y)
Supplement: Supplementary file 4 — Additional file 4: Supplementary Texts 1A-3B. [file 13073_2023_1199_MOESM4_ESM.docx]

**Supplementary Text**

Table of Contents

1 – Supplementary Methods

1A – Confirming genotypes

1B – Calculating incidence

2 – Supplementary Results

2A – Comparison of inferred incidence with known incidence of screened IEMs

2B – Reliability of genotypes

2C – Replication of major results

2D – Consideration of disorder-specific MAF thresholds for BA1

2E – Variants that may not fit the typical screened IEM model

2F – Discussion of gnomAD analysis

2G – Variants shared by both ClinVar and HGMD

3 – Example Calculations

3A – Disease incidence

3B – Reclassifications per variant-month

4 – References

1 – Supplementary Methods

**1A – Confirming genotypes**

To confirm that the inferred pathogenic genotypes we observed in 1KGP were not sequencing errors, we attempted to confirm the quality of all variants that comprised these genotypes (Table S3). Specifically, we downloaded whole genome and deep exome sequencing BAM alignment files of select individuals with homozygous, hemizygous, or compound heterozygous inferred pathogenic genotypes. Most of these alignments were improved by quality-control steps including marking duplicates, local realignment around indels, and base quality recalibration, especially for the Illumina sequencing data. Next, we detected variants and calculated genotypes for each sample at specific sites based on both low-coverage genome sequencing data (<5×per site per individual) and high-coverage exome sequencing information (at least >20×per site per individual) using ‘UnifiedGenotyper’ from the Genome Analysis Toolkit (GATK 3.4-0) under a multi-sample calling strategy(1, 2). Variant Quality Score Recalibration (VQSR) was conducted to evaluate variant quality by GATK 3.4-0. Finally, we obtained variant and genotype information of select individuals and their site-specific genotype quality parameters such as genotype quality (GQ) to validate the quality of the called genotypes. We used GQ≥30 (p-value of 0.001) as our threshold for high quality genotype calls. Genotypes of some individuals (Table S3) were re-confirmed based on high-coverage whole genome sequencing by Complete Genomics(3). Thanks to the recent availability of high-coverage whole genome sequencing of all 1KGP samples from the New York Genome Center(4), the remaining inferred pathogenic genotypes were confirmed using these data. Two inferred pathogenic genotypes in PRODH were not able to be reconfirmed due to poor sequencing quality in the gene.

**1B – Calculating incidence**

To infer screened IEM incidence from 1KGP, for each IEM gene $g$, we summed the allele frequencies of all classified pathogenic variants in $g$, which we call $p_{g}$. Genes were then divided into two categories: X chromosome and autosomal. The disease incidence for all X-linked disorders was calculated as $\sum_{g\in X} p_{g}\left( 1-p_{g} \right)+{p_{g}}^{2}$ where *X* is the set of all X-linked screened IEM genes. For all autosomal genes, the incidence was calculated as $\sum_{g\in A} {p_{g}}^{2}$ where *A* is the set of all autosomal screened IEM genes. An example calculation is provided in Supplementary Text 3A. We repeated this process for each population using the 1KGP population-specific MAF as well. In Figs. S3-5, the height of each bar represents the incidence inferred using the database-wide MAF, while the proportion of the bar comprised by each ancestry is based on the relative disease incidence calculated using the population-specific MAF. The same process was repeated for our gnomAD analysis in Figs. S8-10

2 – Supplementary Results

**2A – Comparison of inferred incidence with known incidence of screened IEMs**

We sought to characterize the extent of misclassified rare variants that could not be removed by a MAF filter or identified as part of an inferred pathogenic genotype. To do this, we compared the screened IEM incidence inferred from each database with the known incidence of screened IEMs. The aggregate incidence of screened IEMs is estimated to be 1 in 3,200 births(5). This includes a small number of X-linked IEMs, which are extremely rare, with an estimated aggregate incidence of 1 in 450,000 births. We used these values as baselines to compare with the inferred incidence of screened IEMs. We inferred the screened IEM incidence of each database from the 1KGP MAF of variants classified as pathogenic (see Supplementary Methods), after applying the 2018 BA1 guidelines. Since the inferred incidence of X-linked IEMs is primarily determined by hemizygous males, we consider autosomal and X-linked IEMs separately. For autosomal IEMs, we found that both Full and Select ClinVar variants inferred an incidence greater than the known incidence prior to 2018 (Fig. S3A,C). By 2018, the inferred incidence fell below the known incidence, and has remained at 20% of the known incidence for both datasets. For X-linked IEMs, Select ClinVar variants have indicated an incidence of zero since 2014 (Fig. S3B). However, Full ClinVar variants have always suggested an incidence orders of magnitude higher than the known incidence, although since 2017 this has been due to just a single variant which primarily is found in East Asian ancestry (Fig. S3D). The more comprehensive perspective provided by screened IEM incidence also allows us to observe patterns that were too subtle to be seen in our analysis of indicated affected individuals. For example, we observed that a large fraction of the screened IEM incidence was skewed towards European ancestry from 2015 to 2017 in Select ClinVar variants (Fig. S3A). However, due to the extreme rarity of these conditions, it is difficult to precisely infer incidence from 1KGP.

When we considered Select HGMD autosomal variants, we found that the inferred screened IEM incidence has decreased slightly over time, yet in 2020 is triple the known incidence (Fig. S4A). The incidence inferred from Full HGMD autosomal variants has increased over time, and in 2020 was 10-fold greater than the known incidence (Fig. S4C). As with Full ClinVar variants, the X-linked IEM incidence suggested by Select and Full HGMD variants is orders of magnitude higher than the known incidence (Fig. S4B,D). The separation of autosomal and X-linked IEMs suggests that African ancestry skew remains among Full HGMD autosomal variants (Fig. S4C), but in our analysis of indicated affected individuals (Fig. S2D) this African ancestry skew is largely masked by X-linked variants with high MAF.

**2B – Reliability of genotypes**

To ensure that the inferred pathogenic genotypes we observed in 1KGP were not caused by errors from variant and genotype calling, we independently confirmed nearly all Select ClinVar variants, Full ClinVar variants, and Select HGMD variants present in an inferred pathogenic genotype (Table S3). We re-called a subset of these genotypes using available low-coverage genome sequencing and high-coverage exome sequencing data from 1KGP (see Methods). We found that nearly all variants classified as pathogenic in this subset passed variant quality score recalibration (VQSR) filtering, and most genotypes in the indicated affected individuals had a genotype quality (GQ) larger than 30. For variants that we did not attempt to re-call or for which re-call quality was poor, we confirmed genotypes using high-coverage whole genome sequencing by either Complete Genomics or the New York Genome Center (see Methods). Out of the entire set of 52 genotypes indicated as pathogenic, there were just two for which genotype quality was below 30. One was in TAZ (NM_000116.5:c.383T>C), present in Select HGMD variants and found in a hemizygous state in HG03196. The other genotype consisted of a pair of compound heterozygous variants in PRODH (NM_016335.6:c.1357C>T;c.1322T>C), present in Full ClinVar variants and harbored by NA19372. Overall, we confirmed that 96% of the inferred pathogenic genotypes are high quality and reproducible. This suggests that the over-representation of putatively pathogenic genotypes in 1KGP is unlikely to be explained by errors introduced by sequencing or data processing.

**2C – Replication of major results**

We replaced 1KGP with gnomAD v3.0 genomes to assess the reproducibility of our major findings. We considered gnomAD individuals from the five continental ancestries (African, Latino, East Asian, European, and South Asian; n = 63,269). gnomAD v3.0 does not include any individuals sampled in 1KGP. However, gnomAD does include individuals enrolled in common disease genetic studies. Additionally, gnomAD does not provide individual-level data, so we were unable to identify compound heterozygous variants. These are significant limitations that restrict our confidence in absolute values derived from this analysis. Instead, we focus on robust claims that can be made from trends over time in the relative values we obtained.

For each cataloged variant, we recorded the number of homozygotes and hemizygotes in gnomAD. Overall, our gnomAD analysis replicated all major findings from our 1KGP analysis (Figs. S6-S10). Across both ClinVar and HGMD, we found that the proportion of individuals in gnomAD that were indicated affected was almost always less than the proportion indicated affected in 1KGP, but not by less than 50%. One exception was the number of gnomAD individuals indicated affected by Full and Select HGMD variants, which was one third of the size expected based on our 1KGP analysis (Fig. S6E,F).

The direction of change in indicated affected individuals for all four datasets over time was nearly always consistent with our 1KGP analysis. We found one notable difference when we considered indicated affected individuals using Select ClinVar variants. In 2015 and 2016, we found an unexpectedly large number of indicated affected individuals (Fig. S6C). This can be attributed to a single variant in *ACADS* (NM_000017.4:c.511C>T) with a gnomAD MAF >3% and which was P with 1 review star in 2015 and 2016. Due to its modest size, 1KGP did not contain any individuals indicated affected by this variant, although the existence of such individuals was suggested by our incidence analysis in 1KGP, which found elevated European ancestry incidence in Select ClinVar variants from 2015 to 2017 (Fig. S3A). The variant, which was classified as P with 1 star in 2015 and 2016, is more prevalent in European ancestry individuals, resulting in European ancestry individuals significantly (p < 3.8×10^-10^) over-represented in 2015 and 2016 Select ClinVar variants, with an odds ratio of 4.0 (95% CI:2.4-6.7). When considering Full ClinVar variants, both East Asian (p < 4×10^-6^) and European (p < 1×10^-4^) ancestry individuals were significantly over-represented from 2014 through 2016, with East Asian individuals having an OR of 4.5 (95% CI:2.6-7.6). These results were obtained by applying the 2015 BA1 guidelines, and they were unaltered when the 2018 BA1 guidelines were applied.

In addition to confirming the African ancestry skew in indicated affected individuals in our 1KGP analysis of HGMD variants, we discovered significant ancestry skew (p < 2×10^-6^) towards East Asian ancestry individuals in Full HGMD variants in 2014 and 2016 (Fig. S6F), as well as significant skew towards European ancestry individuals (p < 2×10^-5^) in Select HGMD variants in 2020 (Fig. S6E). When 2018 BA1 guidelines were applied, significant skew remained for East Asian and European ancestry individuals (Fig. S7E,F). Due to the imbalanced ancestry composition of gnomAD, the described ancestry skew is not obvious from visual inspection of the figures.

With the greater number of individuals in gnomAD relative to 1KGP, we were able to directly compare the false positive rate of Select and Full ClinVar variants. Although there were fewer gnomAD individuals indicated affected by Select ClinVar variants, when considering the indicated affected individuals per cataloged variant, we found that there was little difference between Select and Full ClinVar variants (Fig. S6G).

**2D – Consideration of disorder-specific MAF thresholds for BA1**

In addition to the 2018 BA1 guidelines, Whiffin et al.(6) have also proposed disorder-specific MAF thresholds for BA1 which are supported by recent ACMG/AMP guideline specifications(7). For example, under this system PAH variants would have a stand-alone benign MAF threshold of 1.5% assuming a maximum incidence of 1 in 5,000 births. However, we decided not to pursue Whiffin et al. thresholds due to the heterogeneity of our disorders and complications arising from incomplete penetrance in some disorders. Additionally, many screened IEMs are significantly more common in one ancestry group, due to founder effects, which makes it difficult to define thresholds.

**2E – Variants that may not fit the typical screened IEM model**

In our review of the ClinVar and HGMD variants that imply pathogenic genotypes in 1KGP, we observed some that are perhaps most likely benign, but could possibly be pathogenic, albeit in a way that does not fit the typical phenotype model for screened IEM variants, which generally result in severe, highly-penetrant disorders that begin in infancy or early childhood(8). We note them in an effort to give the benefit of the doubt to the databases. We discovered inferred pathogenic genotypes in 1KGP that included NM_014384.3:c.512C>G in *ACAD8* (associated with asymptomatic disease) and NM_000531.6:c.148G>A in *OTC* (observed in an individual with late onset disease). Asymptomatic IEMs occur when a proband does not have any noticeable signs of disease, but their metabolites reveal a disease phenotype. These variants are generally classified as disease-causing due to their potential to cause disease. However, some individuals will be predicted to have a disease, even though they may never develop symptoms (asymptomatic disease, incomplete penetrance) or symptoms may appear much later in life (late-onset). It is possible that some of these variants do cause symptomatic disease in some individuals but disease has not manifested in the 1KGP individuals.

**2F – Discussion of gnomAD analysis**

Our gnomAD analysis supported the major findings or our 1KGP analysis. However, we noted a persistent issue in which the number of indicated affected gnomAD individuals was proportionally about half of that expected from our 1KGP results. This is potentially explained by our inability to identify compound heterozygotes. In IEM cohorts, a majority of pathogenic genotypes are caused by compound heterozygous variants(9). However, in our 1KGP analysis, compound heterozygotes rarely exceeded 20% of indicated affected individuals, possibly due to false positives with a high allele frequency that contributed to a disproportionately large number of homozygotes. Alternatively, the reduction in gnomAD indicated affected individuals may be caused by the imbalanced ancestry composition of gnomAD, specifically the large fraction of European genomes (which had few indicated affected individuals in our 1KGP analysis) compared with the relative paucity of South Asian or East Asian genomes (which contributed to a large fraction of the indicated affected 1KGP individuals). Despite gnomAD’s imbalanced ancestry composition, its greater size did allow us to compare the false positive rate of Select and Full ClinVar, suggesting that the false positive rate was similar for both datasets. Additionally, our gnomAD analysis revealed ancestry skew towards East Asian and European individuals in both ClinVar and HGMD that could not be definitively detected by 1KGP.

**2G – Variants shared by both ClinVar and HGMD**

We observed a single variant in *ACADS* (NM_000017.4:c.1108A>G) that led to an inferred pathogenic genotype in 1KGP that was present in both the Select ClinVar and Select HGMD datasets. 6 variants that led to an inferred pathogenic genotype were shared by the Full ClinVar and Select HGMD datasets. A total of 8 variants that led to an inferred pathogenic genotype were shared by the Full ClinVar and Full HGMD datasets. By the end of 2020, 7 of these 8 variants were reclassified to a non-pathogenic category in ClinVar, while in HGMD, 4 of the variants were classified as DM, and 4 were classified as DM?

3 – Example Calculations

**3A – Disease Incidence**

Imagine we have the disease-associated Mendelian recessive genes *red*, *orange*, *green* and *blue*. *red* and *orange* are X-linked genes. Each gene has two pathogenic variants cataloged, Var1 and Var2. *red* has redVar1 and redVar2, *orange* has orangeVar1 and orangeVar2, and so on. For one population in 1KGP, the following allele frequencies have been observed:

redVar1: 0.0002

redVar2: 0.0004

orangeVar1: 0.0001

orangeVar2: 0.0003

greenVar1: 0.001

greenVar2: 0.002

blueVar1: 0.005

blueVar2: 0.003

We assume that if one copy of a gene has *any* pathogenic variant AND the other copy of the same gene has *any* pathogenic variant, this will result in a pathogenic genotype (except for hemizygous X-linked genotypes, where *any* pathogenic variant in a single allele is sufficient to create a pathogenic genotype). Thus, we are interested in the sum of allele frequencies of all pathogenic variants for each gene. We call this value $p_{g}$, where $g$ is the gene.

$$p_{red} = 0.0002 + 0.0004 = 0.0006$$

$$p_{orange} = 0.0001 + 0.0003 = 0.0004$$

$$p_{green} = 0.001 + 0.002 = 0.003$$

$$p_{blue} = 0.005 + 0.003 = 0.008$$

Let’s first consider the X-linked genes *red* and *orange*. We want to calculate the incidence of pathogenic genotypes in each gene, and then sum these incidences. We express this mathematically as $\sum_{g\in X} p_{g}\left( 1-p_{g} \right)+{p_{g}}^{2}$ where *X* is the set of X-linked genes. The first term, $p_{g}\left( 1-p_{g} \right)$, is the chance of a hemizygous pathogenic genotype. The second term, ${p_{g}}^{2}$, is the chance of a homozygous or compound heterozygous pathogenic genotype. In this case:

$\sum_{g\in X} p_{g}\left( 1-p_{g} \right)+{p_{g}}^{2}=p_{red}\left( 1-p_{red} \right)+p_{red}^{2}+p_{orange}\left( 1-p_{orange} \right)+p_{orange}^{2}$

$=0.0006\left( 1-0.0006 \right)+{0.0006}^{2}+0.0004\left( 1-0.0004 \right)+\left( 0.0004 \right)^{2}$

$=0.001$

Next, we consider the autosomal genes green and blue. Again, we calculate the incidence of pathogenic genotypes in each gene, and then sum these incidences, which we write as $\sum_{g\in A} {p_{g}}^{2}$ where *A* is the set of all autosomal screened IEM genes. Since pathogenic genotypes can only occur as compound heterozygotes or homozygotes in autosomal genes, we only have a single term, ${p_{g}}^{2}$. In our example:

$\sum_{g\in A} {p_{g}}^{2}=p_{green}^{2}+p_{blue}^{2}={0.003}^{2}+{0.008}^{2}=0.000073$

In conclusion, in this example we found a X-linked disease incidence of 0.001 (very high!), and an autosomal disease incidence of 0.000073 (more reasonable).

**3B – Reclassifications per variant-month**

Imagine we have the following classification history for variants in ClinVar for a certain ancestry:

|  | May | June | July |
| --- | --- | --- | --- |
| Var1 | Pathogenic | VUS | VUS |
| Var2 | Pathogenic | Pathogenic | Pathogenic |
| Var3 | Pathogenic | Pathogenic | Pathogenic |

The only reclassification during this three-month period was Var1, which was reclassified from Pathogenic to VUS in June. Thus, the number of reclassifications was 1. To normalize for the fact that some ancestries have more cataloged variants than others, we consider how many variants had the opportunity to be reclassified, and for how long that opportunity lasted. We measure this combination of both quantity and time as variant-months. In the table above, each cell represents a single variant-month. Thus, there were 7 variant-months of Pathogenic, and 2 variant-months of VUS. If we are considering reclassification in the direction of decreasing confidence (Pathogenic to VUS), we are only interested in the 7 variant-months of Pathogenic, since VUS variants cannot be reclassified to a less confident category. To calculate reclassifications per variant-month, we simply divide 1 reclassification by 7 variant-months to reach 0.14 reclassifications per variant-month.

4 – References

1. DePristo MA, Banks E, Poplin R, Garimella KV, Maguire JR, Hartl C, et al. A framework for variation discovery and genotyping using next-generation DNA sequencing data. Nat Genet. 2011;43(5):491-8.

2. Van der Auwera GA, Carneiro MO, Hartl C, Poplin R, Del Angel G, Levy‐Moonshine A, et al. From FastQ data to high‐confidence variant calls: the genome analysis toolkit best practices pipeline. Current protocols in bioinformatics. 2013;43(1):11.0. 1-.0. 33.

3. Drmanac R, Sparks AB, Callow MJ, Halpern AL, Burns NL, Kermani BG, et al. Human genome sequencing using unchained base reads on self-assembling DNA nanoarrays. Science. 2010;327(5961):78-81.

4. Byrska-Bishop M, Evani US, Zhao X, Basile AO, Abel HJ, Regier AA, et al. High coverage whole genome sequencing of the expanded 1000 Genomes Project cohort including 602 trios. bioRxiv. 2021.

5. Feuchtbaum L, Carter J, Dowray S, Currier RJ, Lorey F. Birth prevalence of disorders detectable through newborn screening by race/ethnicity. Genet Med. 2012;14(11):937-45.

6. Whiffin N, Minikel E, Walsh R, O’Donnell-Luria AH, Karczewski K, Ing AY, et al. Using high-resolution variant frequencies to empower clinical genome interpretation. Genet Med. 2017;19(10):1151-8.

7. Harrison SM, Biesecker LG, Rehm HL. Overview of specifications to the ACMG/AMP variant interpretation guidelines. Current protocols in human genetics. 2019;103(1):e93.

8. Adhikari AN, Currier RJ, Tang H, Turgeon CT, Nussbaum RL, Srinivasan R, et al. Genomic Analysis of Historical Cases with Positive Newborn Screens for Short-Chain Acyl-CoA Dehydrogenase Deficiency Shows That a Validated Second-Tier Biochemical Test Can Replace Future Sequencing. International journal of neonatal screening. 2020;6(2):41.

9. Blau N. Genetics of phenylketonuria: then and now. Hum Mutat. 2016;37(6):508-15.
