# Supplementary material for: ClinVar and HGMD genomic variant classification accuracy has improved over time, as measured by implied disease burden
Source: Genome Med. 2023 Jul 13;15:51. doi: 10.1186/s13073-023-01199-y (PMC10347827; doi:10.1186/s13073-023-01199-y)
Supplement: Supplementary file 6 — Additional file 6: Table S4. ClinVar variants found in a pathogenic genotype in one or more predicted affected individuals from 1KGP. [file 13073_2023_1199_MOESM6_ESM.docx]

| Chr | Position | Ref | Alt | Gene | cDNA,  protein | Variant type | # hom or hemi in 1KGP | # comp het in 1KGP | 1KGP sample ID(s) | First pathogenic submission: Submitter, Date, Interpretation, Evidence | First non-pathogenic submission: Submitter, Date, Interpretation, Evidence | Consensus Interpretation as of Dec 2020* | Submitted  Interpretations | |
| --- | --- | --- | --- | --- | --- | --- | --- | --- | --- | --- | --- | --- | --- | --- |
| 5 | 132384290 | C | T | SLC22A5 | c.713C>T  p.A238G | missense | 1 | 0 | HG04075 | Research and Development (ARUP Laboratories),  April 2014,  Pathogenic,  Present in affected individuals | EGL Genetic Diagnostics,  January 2017,  VUS,  Clinical testing | Conflicting Interpretations of Pathogenicity | 1 Pathogenic  3 Likely pathogenic  1 VUS |  |
| 6 | 49456101 | G | A | MMUT | c.890C>T  p.T297I | missense | 2 | 0 | HG03598  HG03686 | Institute of Medical Genetics and Genomics (Sir Ganga Ram Hospital), October 2018, Pathogenic, None | Invitae, December 2019, Likely benign, Clinical testing | Conflicting Interpretations of Pathogenicity | 1 Pathogenic NC 1 VUS, 1 Likely benign 1 Benign NC |  |
| 9 | 130452225 | C | T | ASS1 | c.-4=  NA | 5' UTR | 0 | 1 | NA19030 | GeneDx, August 2015, Pathogenic, clinical testing | Invitae, January 2017, VUS, Non-coding variant seen in affected and in population databases | Conflicting Interpretations of Pathogenicity | 2 Likely pathogenic 2 VUS |  |
| 9 | 130458549 | G | T | ASS1 | c.323G>T  R108L | missense | 1 | 1 | NA19030  NA19395 | OMIM,  April 2014,  Pathogenic,  Heterozygous variant in affected individual | Illumina,  April 2017,  VUS,  Observed in healthy population | Conflicting Interpretations of Pathogenicity | 1 Pathogenic NC 1 VUS 4 Likely benign 1 Benign 1 Benign NC |  |
| 12 | 109561798 | C | T | MMAB | c.403G>A  p.A135T | missense | 1 | 0 | HG03169 | GeneReviews, February 2016, Pathogenic, Seen in affected individuals | GeneDx, May 2017, VUS, Variant is conserved and predicted damaging, but seen at high frequency in African ancestry populations. | Conflicting Interpretations of Pathogenicity | 1 Pathogenic NC 1 VUS 1 Benign 1 Benign NC |  |
| 12 | 120739317 | A | G | ACADS | c.1108A>G  p.M370V | missense | 1 | 0 | NA20878 | GeneDx, August 2015, Pathogenic, clinical testing | Illumina, January 2017, VUS, Observed in both healthy and affected individuals, with high population allele frequency | Conflicting Interpretations of Pathogenicity | 2 VUS 1 Likely Benign |  |
| 22 | 18918386 | G | A | PRODH | c.1357C>T  p.R453C | missense | 0 | 1 | NA19372 | OMIM,  April 2014, Pathogenic, In vitro assay | Laboratory for Molecular Medicine (Partners HealthCare Personalized Medicine), May 2017,  Benign, Elevated population allele frequency | Conflicting Interpretations of Pathogenicity | 1 Pathogenic NC 1 VUS 1 Likely benign 1 Benign |  |
| 22 | 18918421 | A | G | PRODH | c.1322T>C  p.L441P | missense | 0 | 1 | NA19372 | OMIM, April 2014, Pathogenic, In vitro assay | Invitae, December 2017, VUS, Highly conserved, with in vitro evidence, but high allele frequency | Conflicting Interpretations of Pathogenicity | 1 Pathogenic NC 3 Likely pathogenic 1 VUS |  |
| X | 38367361 | G | A | OTC | c.148G>A  p.G50R | missense | 1 | 0 | NA21124 | GenMed Metabolism Lab, April 2014, Pathogenic, Identified in late onset individual | None | Pathogenic, 0 stars^†^ | 2 Pathogenic NC |  |
| X | 38369882 | G | C | OTC | c.298+5G>C  NA | Donor splice site | 3 | 0 | HG00622  HG01844  HG02073 | GenMed Metabolism Lab, April 2014, Pathogenic, Identified in affected female | EGL Genetic Diagnostics, January 2017, Benign Classified in clinical testing | Benign/Likely benign | 1 Pathogenic NC 1 VUS NC 1 Likely benign 5 Benign 1 Benign NC |  |
| X | 38381417 | C | T | OTC | c.374C>T  p.T125M | missense | 1 | 0 | NA19117 | GenMed Metabolism Lab, April 2014, Pathogenic, Identified in affected individual | University of Washington,  January 2015, VUS, Classified as part of investigation of incidental findings in population cohort | VUS | 3 VUS 1 Pathogenic NC |  |

**Table S6.** ClinVar variants found in a pathogenic genotype in one or more predicted affected individuals from 1KGP. *ClinVar only considers variants with criteria in determining consensus interpretation. NC = No assertion criteria provided. ^†^As of May 2022, this variant was classified as Conflicting Interpretations of Pathogenicity.

The exact transcript versions that we used for the HGVS notation in the above genes

SLC22A5: NM_001308122.2, NP_001295051.1

MMUT: NM_000255.4, NP_000246.2

ASS1: NM_000050.4, NP_000041.2

MMAB: NM_052845.4, NP_443077.1

ACADS: NM_000017.4, NP_000008.1

PRODH: NM_016335.6, NP_057419.5

OTC: NM_000531.6, NP_000522.3
